# Position‐Scanning Peptide Libraries as Particle Immunogens for Improving CD8^+^ T‐Cell Responses

**DOI:** 10.1002/advs.202103023

**Published:** 2021-10-30

**Authors:** Xuedan He, Shiqi Zhou, Breandan Quinn, Wei‐Chiao Huang, Dushyant Jahagirdar, Michael Vega, Joaquin Ortega, Mark D. Long, Fumito Ito, Scott I. Abrams, Jonathan F. Lovell

**Affiliations:** ^1^ University at Buffalo State University of New York Buffalo NY 14260 USA; ^2^ Department of Anatomy and Cell Biology McGill University Montreal Quebec H3A1Y2 Canada; ^3^ Division of Research and Innovation Partnerships Northern Illinois University DeKalb IL 60115 USA; ^4^ Department of Cancer Genetics and Genomics Roswell Park Comprehensive Cancer Center (RPCCC) Buffalo NY 14263 USA; ^5^ Department of Immunology Roswell Park Comprehensive Cancer Center Buffalo NY 14263 USA; ^6^ Center for Immunotherapy Roswell Park Comprehensive Cancer Center Buffalo NY 14263 USA; ^7^ Department of Surgical Oncology Roswell Park Comprehensive Cancer Center Buffalo NY 14263 USA

**Keywords:** cancer, immunogen, liposomes, position‐scanning peptide libraries, vaccines

## Abstract

Short peptides reflecting major histocompatibility complex (MHC) class I (MHC‐I) epitopes frequently lack sufficient immunogenicity to induce robust antigen (Ag)‐specific CD8^+^ T cell responses. In the current work, it is demonstrated that position‐scanning peptide libraries themselves can serve as improved immunogens, inducing Ag‐specific CD8^+^ T cells with greater frequency and function than the wild‐type epitope. The approach involves displaying the entire position‐scanning library onto immunogenic nanoliposomes. Each library contains the MHC‐I epitope with a single randomized position. When a recently identified MHC‐I epitope in the glycoprotein gp70 envelope protein of murine leukemia virus (MuLV) is assessed, only one of the eight positional libraries tested, randomized at amino acid position 5 (Pos5), shows enhanced induction of Ag‐specific CD8^+^ T cells. A second MHC‐I epitope from gp70 is assessed in the same manner and shows, in contrast, multiple positional libraries (Pos1, Pos3, Pos5, and Pos8) as well as the library mixture give rise to enhanced CD8^+^ T cell responses. The library mixture Pos1‐3‐5‐8 induces a more diverse epitope‐specific T‐cell repertoire with superior antitumor efficacy compared to an established single mutation mimotope (AH1‐A5). These data show that positional peptide libraries can serve as immunogens for improving CD8^+^ T‐cell responses against endogenously expressed MHC‐I epitopes.

## Graphical abstract

Short peptides usually lack sufficient immunogenicity to induce robust antigen (Ag)‐specific CD8^+^ T‐cell responses. With a next‐generation vaccine adjuvant, it is demonstrated that position‐scanning peptide libraries of tumor antigens themselves can serve as improved immunogens to induce Ag‐specific CD8^+^ T cells with greater frequency and function compared to the wild‐type epitope.

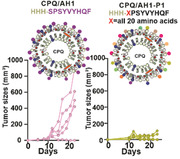

## 1 Introduction

Nanotechnology holds promise to enhance cancer immunotherapy and cancer vaccines.^[^ ^1^, ^2^, ^3^, ^4^, ^5^ ^]^ Peptide vaccines are appealing owing to their simplicity in production and direct targeting of relevant MHC‐I and MHC‐II cancer epitopes.^[^ ^6^, ^7^ ^]^ Mimotopes of T‐cell epitopes are usually peptides derived de novo or from modifying known epitopes.^[^ ^8^ ^]^ For de novo mimotopes, pre‐isolated T‐cell clones can be used to find active amino acid sequences from positional scanning synthetic peptide libraries. These libraries comprise defined amino acids in a single position, while all other positions are substituted randomly with all 20 amino acids or 19 amino acids (excluding cysteine).^[^ ^9^, ^10^ ^]^ For a 9‐mer peptide, such a library would contain 180 individual library compounds to screen, which would be analyzed for key specific residues that induce T cell reactivity.^[^ ^11^, ^12^, ^13^, ^14^, ^15^, ^16^, ^17^ ^]^ However, this in vitro screening approach is laborious and requires CD8^+^ T cell clones in hand. It also does not guarantee that identified peptides will be effective immunogens in vivo. For mimotopes based on already known epitopes, it is possible to substitute amino acids that either enhance the stability of the peptide‐MHC (pMHC) complex,^[^ ^18^, ^19^, ^20^, ^21^ ^]^ or alternatively enhance the pMHC‐T cell receptor binding in such a manner to enhance the T‐cell induction.^[^ ^22^ ^]^ Herein, these are termed enhanced mimotopes or “e‐mimotopes.” For example, the human Melan‐A/MART‐1_26–35_ mutation A27L (E**L**AGIGILTV) was discovered by substituting the position 2 amino acid, a pMHC anchor residue, with a bulky leucine residue to attempt to increase binding to HLA‐A*0201.^[^ ^23^ ^]^ gp100 2M (I**M**DQVPFSV) was discovered using the same approach by substituting amino acids of the wild‐type peptide at the HLA‐A*0201‐binding anchor positions, but not at T‐cell receptor (TCR) contact residues, to increase peptide MHC class I (MHC‐I) binding affinity.^[^ ^24^ ^]^ NY‐ESO‐1 C165V (SLLMWITQ**V**) was discovered by changing cysteinylation and dimerization of cysteine residues to enhance the antigenicity of synthetic peptides binding to MHC‐I molecules.^[^ ^25^ ^]^ The AH1‐A5 (SPSYAYHQF) e‐mimotope (referred to as A5 herein) was discovered when alanine scanning revealed that substitution at the fifth position gave rise to enhanced pMHC‐TCR binding.^[^ ^22^ ^]^ E‐mimotopes have been translated to clinical trials for cancer vaccines, such as the melanoma antigen Melan‐A/MART‐1_26‐35_ A27L,^[^ ^26^, ^27^ ^]^ gp100 2M, NY‐ESO‐1 C165V, and Survivin T97M (ELMLGEFLKL).^[^ ^28^, ^29^ ^]^ Generally, while these e‐mimotopes produced immunogenic responses, like most cancer peptide vaccines in general, there was insufficient reactivity with tumor‐expressing epitopes and thus there remains a substantial challenge to identify more functional peptides for cancer vaccines.

An ideal e‐mimotope should increase the frequency of tumor‐specific T cells, recognize tumor antigens and induce tumor protection after vaccination. The MHC‐I epitope Env_37‐44_ (SPHQVFNL), from the MuLV gp70 envelope protein, was identified recently through genomic and proteomic screening.^[^ ^30^ ^]^ RNAseq data and mass spectrometry‐based immunopeptidome analysis identified that Env_37‐44_ is presented on MHC‐I in the CT26 cancer cell line. This same peptide expression on CT26 cells was confirmed independently by another group with tetramer‐based flow cytometry analysis of T cells, albeit with low expression.^[^ ^31^ ^]^ A similar MHC‐I‐restricted peptide is AH1, also derived from the gp70 (amino acid 423‐431) of MuLV.^[^ ^32^ ^]^ It is highly expressed in certain cancer cells but poorly expressed in the normal cells, making it a tumor rejection Ag in several murine tumor models.^[^ ^22^, ^33^, ^34^ ^]^ Increased expression of gp70 in aged mice has been shown to diminish the capability to induce AH1‐specific CD8^+^ T cells.^[^ ^35^ ^]^ Although CD8^+^ T cells with self‐specific TCRs are deleted in the process of central tolerance, such populations are reduced but not eliminated.^[^ ^36^ ^]^ Nevertheless, AH1‐specific CD8^+^ T cells are not easily induced in mice by vaccination with the wild‐type epitope, but can be induced using mimotopes that have mutations in the wild‐type epitope such as A5 (which has position 5 of AH1 mutated to alanine) or other mutations.^[^ ^37^ ^]^ A5 has been shown to stabilize the pMHC‐TCR complex,^[^ ^22^ ^]^ which is a better predictor than peptide‐MHC‐I affinity for cytotoxic T lymphocyte (CTL) immunogenicity.^[^ ^38^ ^]^

AH1 peptide‐MHC libraries constructed using a recombinant baculovirus‐expressed protein construct have been screened by isolating T cell clones to identify e‐mimotopes, which were then used as vaccine immunogens to induce enhanced anti‐tumor efficacy.^[^ ^39^, ^40^ ^]^ Variable epitope libraries carrying on the order of 1 × 10^5^ mutated epitope variants have been proposed as phage and peptide vaccine immunogens, however also displayed limited antitumor efficacy.^[^ ^41^, ^42^ ^]^ In the current study, we use an alternative approach by using position‐scanning libraries comprising just 20 peptide members per library, and presenting the libraries on highly immunogenic liposomes. Since a single amino acid change of peptide can change the resulting T‐cell response,^[^ ^43^, ^44^, ^45^, ^46^ ^]^ we hypothesized that a positional library vaccine with a randomized position within the peptide sequence might find improvements to the immunogenicity of the wild‐type peptide. Thus, instead of using isolated T‐cell clones as tools to screen randomized peptide libraries, a position scanning library would be used as the immunogen. To make the positional peptide library particle vaccine, CPQ (CoPoP/PHAD/QS‐21) liposomes containing cobalt porphyrin–phospholipid (CoPoP), phosphorylated hexaacyl disaccharide (PHAD), and quillaja saponaria (QS‐21) were used as an adjuvant system to generate immune responses against MHC‐I‐restricted immunogens. PHAD is a synthetic form of monophosphoryl lipid A (MPLA), which is a toll‐like receptor 4 agonist. QS‐21 and MPLA are components of a licensed liposomal vaccine adjuvant AS01.^[^ ^47^ ^]^ CoPoP liposomes contain intrabilayer chelated cobalt which rapidly forms coordinate bonds with peptides bearing an abbreviated polyhistidine‐tag (his‐tag).^[^ ^48^, ^49^, ^50^, ^51^, ^52^ ^]^ CPQ is a strong cancer vaccine adjuvant system for inducing CD8^+^ T‐cell responses based on: 1) codelivery of the MHC‐I binding immunogen together with MPLA and QS21 to antigen‐presenting cells (APCs), 2) improved delivery of the immunogen to APCs following conversion of the peptides into particles, and 3) a putative delivery mechanism that enables direct transfer of the peptide from carrier to MHC‐I in APCs.^[^ ^53^, ^54^ ^]^ In prior studies, we have shown that CPQ induced immune responses capable of inhibiting tumor growth with nanogram antigen dosing. In contrast, 2HPQ (2H/PHAD/QS21), which is identical to CPQ except that the cobalt is replaced with two hydrogen atoms, was not capable of forming peptide particles and did not induce antitumor immune responses. Admixture of epitopes with polyinosinic:polycytidylic acid (poly(I:C)) or aluminum gel (Alum) also did not result in induction of functional immune responses with immunization.^[^ ^53^, ^54^ ^]^ CPQ liposomes have also been shown to be an effective adjuvant system for multiplexing as many as 20 antigens to induce CD8^+^ T‐cell responses.^[^ ^53^, ^54^ ^]^

In this study, using two known MHC‐I epitopes, vaccines are formed by positional scanning peptide libraries which were converted into particle form with CPQ liposomes. Both target MHC‐I epitopes became substantially more immunogenic with a positional library approach, with the random amino acid being set at specific positions. We found an Env_37‐44_ positional peptide library with amino acid substitution on position 5 induced high levels of Ag‐specific CD8^+^ T cells compare to wild‐type Env_37‐44_ peptide and these induced T cells could cross react with wild‐type peptide. We also identified the active amino acid on position 5 is Alanine by screening upon vaccination. These T cells induced by Env_37‐44_‐A5 lysed CT26 cells in vitro but did not inhibit tumor growth in mice. The positional library vaccine strategy also worked for the AH1 epitope, especially the multivalent positional library vaccine, which eradicated established tumor more efficiently. The multivalent peptide library vaccine also elicited T cells with higher affinity with AH1 tetramer and more cytokine‐producing CD8^+^ T cells than the wild‐type peptide AH1.

## 2 Results and Discussion

### 2.1 Env_37‐44_ Positional Peptide Libraries Form Particles When Admixed with CPQ Liposomes

**Figure 1:**
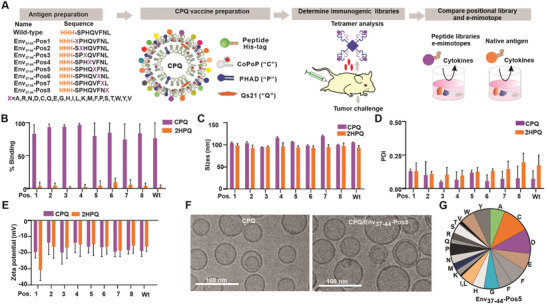
Env_37‐44_ positional peptide libraries as particle immunogens. A) Scheme of experimental design of demonstrating positional library as efficient immunogens. B) Percentage of Env_37‐44_ whole peptide libraries that bind to CPQ or 2HPQ liposomes. C) Sizes, D) polydispersity, and E) zeta potential of CPQ and 2HPQ liposomes after binding with libraries. F) Cryo‐electron micrographs of CPQ liposomes with or without the Env_37‐44_ ‐Pos5 positional peptide bound. Representative images from a single experiment are shown. G) Approximate peptide distribution of the Env_37‐44_ ‐Pos5 positional library. Error bars show mean ± std. dev. for *n* = 3 triplicate experiments.

Position‐scanning libraries were synthesized as shown in **Figure** **1**A. Three histidines were added to the N‐terminus of all peptides and peptide libraries for particle formation with CoPoP liposomes. We previously found that his‐tag length can cause minor interference in synthetic epitope binding to H‐2L^d^, although this did not significantly impact the induction of Ag‐specific CD8^+^ T cells in mice.^[^ ^53^ ^]^ For each library, the amino acid at a specific position was substituted with all 20 amino acids while the remainder of the positions were kept the same as the wild‐type sequence. For this 8‐mer peptide, we made 8 positional libraries. Peptides were combined with CPQ to form a single peptide vaccine or positional peptide library vaccine for mice. The immunogenicity of these immunogens was assessed by wild‐type antigen Env_37‐44_ tetramer staining; and the antitumor efficacy of these immunogens was assessed by challenging mice with CT26 cells subcutaneously. The cross‐reactivity of T cells induced by positional peptide libraries and e‐mimotopes to native peptides was evaluated by cytokine production after in vitro Ag stimulation of splenocytes.

CPQ liposomes were used for the library immunogen delivery platform. All positional peptide libraries and the wild‐type Ag are bound to the CPQ liposome, but not the 2HPQ liposome which has identical components as CPQ but has cobalt replaced in the porphyrin macrocycle by two hydrogens (Figure 1B). We did not assess the binding of individual library members, but rather the overall pool of peptides as a whole. The size of CPQ and 2HPQ liposomes remained ≈100 nm after peptide binding (Figure 1C) and the polydispersity (PDI) of liposomes after remained lower than 0.3 (Figure 1D). There was no obvious difference in zeta potential between CPQ and 2HPQ, which was all negative (Figure 1E). Cryo‐electron microscopy revealed that both CPQ liposomes with or without peptide bound were spherical, with a diameter close to 50–90 nm (Figure 1F). These data indicated that CPQ liposomes displaying the peptide libraries were well‐formed nanoparticles free from aggregation; however, further research is required to understand the discrepancy in size measurement between these two methodologies. In general, however, light scattering can be biased towards larger size particles, whereas, cryo‐electron microscopy should reflect actual morphology and dimensions.^[^ ^55^, ^56^ ^]^ Liquid chromatography‐mass spectrometry (LC‐MS) was used to assess the peptide distribution of positional libraries (Figure S1 and Table S1, Supporting Information). Based on the peak area of each peptide in the library, the distribution of each peptide in the Env_37‐44_‐Pos5 appeared relatively random, as expected, although some amino acids were more represented than others (Figure 1G). The median peptide distribution percentage is 4.8% and the interquartile range is 2.4%–6.9%. However, more than 85% of the peptides expressed over 2% in the peptide library. Despite these limitations, peptides synthesized in the positional library are much more convenient than individual synthesis and pooling of 20 separate peptides.

### 2.2 The Immunogenicity of Env_37‐44_ Positional Peptide Libraries

**Figure 2:**
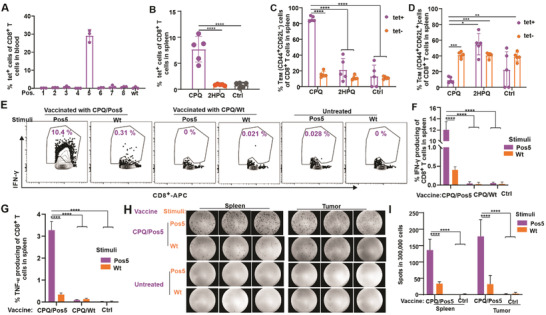
CD8^+^ T cells responses induced by the Env_37‐44_‐Pos5 positional library particle vaccine. BALB/c mice were untreated or vaccinated with CPQ and indicated peptide libraries on days 0 and 7, then blood was collected on day 14. A) Percentage of Env_37‐44_ tet^+^ cells in the CD8^+^ T cell population in blood. Error bars show mean ± std. dev. for *n* = 3 mice per group. BALB/c mice were untreated or vaccinated on days 0 and 7, then splenocytes were prepared on day 14. B) Percentage of Env_37‐44_ tet^+^ cells in the CD8^+^ T‐cell population in spleen. C) T_EM_ (CD44^+^CD62L^–^) cells or D) T_CM_ (CD44^+^CD62L^+^) in the Env_37‐44_ tet^+^ CD8^+^ T cell or Env_37‐44_ tet^–^ CD8^+^ T cell population. Error bars show mean ± std. dev. for *n* = 5 mice per group. E) Flow cytometry gating and F) percentage of IFN‐*γ* and G) TNF‐*α* producing cells in the CD8^+^ T cell population after 10 µg mL^–1^ Ag stimulation. Error bars show mean ± std. dev. for *n* = 3 mice per group. BALB/c mice were inoculated with CT26 tumor on day 0, then vaccinated on day 8 and 15; splenocytes and tumor infiltrating lymphocytes (TIL) were prepared on day 21 for analysis. Splenocytes or TIL from CPQ/Env_37‐44_‐Pos5 vaccinated mice and untreated mice were stimulated with Pos5 library and wild‐type peptide for 24 h. H) Images of ELISpot results. Images show results of three biological replicates. I) Summary of the ELISpot results. Error bars show mean ± std. dev. for *n* = 3 mice per group. * *p* < 0.05, ** *p* < 0.01, *** *p* < 0.001, and **** *p* < 0.0001, analyzed by (B, C, D, F, G, I) one‐way ANOVA with Tukey multiple comparisons post‐test.

The NetMHC neural network algorithm was used to predict the binding affinity of each positional library.^[^ ^57^, ^58^ ^]^ The H‐2L^d^, H‐2D^d^ and H‐2K^d^ binding percentiles of wild‐type Env_37‐44_ were 0.015%, 2.5%, and 17%, respectively; thus, Env_37‐44_ represents an excellent H‐2L^d^ MHC‐I binder. The individual library members of Env_37‐44_ ‐Pos5 were predicted to have, in general, slightly better binding with H‐2L^d^ compared to the native epitope (Figure S2, Supporting Information). We next used the 8 positional peptide library particles to immunize mice and compare immunogenicity to the native Env_37‐44_ vaccine. In all cases, the peptides were admixed with CPQ liposomes to induce particle formation of the peptides. An Env_37‐44_ tetramer was used to assess Env_37‐44_ specific CD8^+^ T cells. BALB/c mice were immunized on days 0 and 7; then blood was collected on day 14 for tetramer analysis. Of all the positional libraries assessed, only CPQ/Env_37‐44_‐Pos5 vaccine induced 30% of CD8^+^ T cells that were Env_37‐44_ specific. In contrast, other peptide library vaccines or the wild‐type epitope vaccine did not elicit any detectable Env_37‐44_ specific CD8^+^ T cells (**Figure** **2**A). The Pos5 library was admixed with CPQ or 2HPQ liposomes and injected intramuscularly into BALB/c mice on days 0 and 7, and splenocytes were isolated on day 14 for immune analysis. Based on the flow cytometry gating (Figure S3, Supporting Information), mice vaccinated with CPQ/Pos5 produced ≈7% of tet^+^ cells in the CD8^+^ T cell population in the spleen; however, the nonparticle forming liposome 2HPQ with Pos5 library did not show any difference compared to the untreated control group (Figure 2B). With the CPQ vaccination, ≈80% and ≈10% tet^+^ CD8^+^ T cells expressed an effector‐memory phenotype (T_EM_) (CD62L^–^ CD44^+;^ Figure 2C) or central‐memory phenotype (T_CM_) (CD62L^+^ CD44^+^; Figure 2D), respectively. In the CPQ vaccine group, the phenotype of tet^+^CD8^+^ and tet^–^CD8^+^ T cells were significantly different; however, the 2HPQ vaccine and untreated groups showed no differences in the phenotype of tet^+^ CD8^+^ and tet^–^ CD8^+^ T cells.

Tetramer staining tends to efficiently detect high‐affinity TCRs, but can potentially neglect low affinity ones even though T cells with low‐affinity TCRs can be significant effectors in the immune response.^[^ ^59^ ^]^ As an alternative to tetramer staining, intracellular IFN‐*γ* staining was carried out on splenocytes from immunized mice by stimulation with Pos5 or wild‐type peptide. Based on the flow cytometry gating (Figure 2E and Figure S4, Supporting Information), splenocytes from untreated mice or mice vaccinated with wild‐type peptides showed no detectable IFN‐*γ* or TNF‐*α* production neither after Env_37‐44_‐Pos5 stimulation nor wild‐type peptide stimulation. Splenocytes from Pos5 vaccinated mice had ≈12% and 3% of CD8^+^ T cells that produced IFN‐*γ* (Figure 2F) and TNF‐*α* (Figure 2G), respectively after Pos5 stimulation. However, only ≈0.5% and ≈0.3% of CD8^+^ T cells produced IFN‐*γ* and TNF‐*α*, respectively after wild‐type peptide stimulation (Figure 2F). This indicates that a higher proportion of CD8^+^ T cells reacted with the Env_37‐44_‐Pos5 library, compared to the wild‐type Env_37‐44_ epitope. An enzyme‐linked immunospot (ELISpot) assay was used to confirm these results in both the spleen and tumor. With the CPQ/Pos5 vaccination, more T cells were induced that responded to Pos5 stimulation, whereas less cells responded to wild‐type peptide stimulation to produce these cytokines in both the spleen and tumor (Figure 2H,I).

To better characterize the Env_37‐44_‐Pos5 positional library, we individually synthesized 20 peptides, each with a different amino acid at position 5 (details shown Table S2, Supporting Information). The concentration of each peptide and peptide library were assessed by the BCA assay (Figure S5, Supporting Information). All 20 peptides from Env_37‐44_‐Pos5 were then individually admixed with CPQ for vaccination (Figure S6A, Supporting Information). Vaccines were administrated on days 0 and 7, then blood was collected for analysis on day 14. Among all of these 20 peptides derived from position 5, one substitution Env_37‐44_‐A5 produced much higher percentages of tetramer‐positive (tet^+^) CD8^+^ T cells in the blood (Figure S6B, Supporting Information) compared to untreated mice. Glycine and Proline substituted at position 5 also produced higher levels of AH1‐tet^+^ CD8^+^ T cells, showing that multiple substitutions at this position hold potential to create e‐mimotopes. In an in vitro cell lysis study, when tumor cells were pulsed with the wild‐type Env_37‐44_ peptide or Env_37‐44‐_A5 e‐mimotope, splenocytes from mice vaccinated with Env_37‐44_‐A5 lysed more than 60% of the tumor cells at an effector to target cell (E:T) ratio of 50:1(Figure S6C,D, Supporting Information). Splenocytes from untreated mice or mice vaccinated with wild‐type peptide did not induce tumor cell‐specific lysis even at the highest E:T ratio tested. However, splenocytes from Env_37‐44_‐A5 e‐mimotope vaccinated mice were not capable of lysing CT26 cells without peptide pulse in vitro (Figure S6E, Supporting Information). Unfortunately, neither CPQ/Env_37‐44_‐Pos5 nor CPQ/Env_37‐44_‐A5 protected mice from tumor challenge (Figure S7, Supporting Information). There are multiple reasons that could account for the lack of anti‐tumor efficacy induced by vaccination with CPQ/Env_37‐44_‐A5 or CPQ/Env_37‐44_‐Pos5. The in vitro CTL experiment showed that pulsing tumor cells with wild‐type Env_37‐44_ or Env_37‐44_‐A5 peptide was essential for splenocytes from CPQ/A5‐vaccinated mice to lyse tumor cells. This suggests there may be insufficient levels of expression of this Ag with MHC‐I on the tumor cell surface. Additionally, the IFN‐*γ* or TNF‐*α* production and ELISpot results indicated that the positional library vaccine‐induced CD8^+^ T cells were not as strongly activated by wild‐type peptide stimulation compared to Env_37‐44_‐Pos5 stimulation. This could signify that the library‐induced CD8^+^ T cells were not sufficiently cross‐reactive with the wild‐type epitope to induce functional anti‐tumor responses.

### 2.3 AH1 Positional Peptide Libraries and Library Mixtures as Immunogens

**Figure 3:**
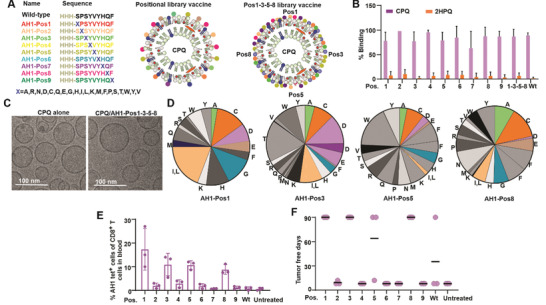
AH1‐Pos1, AH1‐Pos3, AH1‐Pos5, and AH1‐Pos8 library vaccine immunogens induced more AH1‐specific CD8^+^ T cells than the wild‐type epitope and protected mice from tumor challenge. A) Scheme of CPQ vaccines that made of one AH1 positional library or 4 AH1 positional libraries. B) Percentage of AH1 peptide libraries bind to CPQ and 2HPQ liposomes. Error bars show mean ± std. dev. for *n* = 3 triplicate experiments. C) Cryo‐electron micrographs of CPQ and CPQ/AH1‐Pos1‐3‐5‐8. Representative images from a single experiment are shown. D) Amino acid distribution of AH1‐Pos1, AH1‐Pos3, AH1‐Pos5 and AH1‐Pos8 positional library. BALB/c mice were untreated or vaccinated with CPQ and AH1 wild‐type peptide or AH1 peptide libraries on days 0 and 7, then blood was collected for analysis and CT26 tumor cells were inoculated subcutaneously on day 14. E) Percentage of AH1 tet^+^ cells in the CD8^+^ T cell population. Error bars show mean ± std. dev. for *n* = 3. F) Tumor free days. The experiment was performed with *n* = 3 independent mice and the line shows the mean.

Next, we applied the positional library vaccine strategy to the second MuLV epitope; AH1. Unlike Env_37‐44,_ AH1 is an established tumor rejection epitope expressed highly on MHC‐I of CT26 cancer cells. Positional libraries were generated for each amino acid position of the 9‐mer AH1 sequence as shown in **Figure** **3**A. The H‐2L^d^ binding motif of AH1 has been reported to include a proline (P) at position 2 and a phenylalanine (F) on position 9,^[^ ^60^ ^]^ while the tyrosine (Y) residues at positions 4 and 6 are important for TCR recognition.^[^ ^22^ ^]^ Consequently, a slight change in positions 4 and 6 could alter T cell activation. It has been reported that alanine substitution of the peptide on MHC anchor positions 2 and 9 or T cell binding positions 4 and 6 or even position 7 led to no cytolytic activity. Still, substitutions at positions 1, 3, 8 and especially position 5 led to lysis of target cells.^[^ ^22^ ^]^ Therefore, besides positional individual peptide libraries, we also prepared a combined library of libraries; a positional peptide library (AH1‐Pos1‐3‐5‐8) made of AH1‐Pos1, AH1‐Pos3, AH1‐Pos5 and AH1‐Pos8. With a 3 residue his‐tag on the N terminal, all peptide libraries could bind to CPQ liposomes but not 2HPQ liposomes (Figure 3B). Sizes of CPQ and 2HPQ liposome remained ≈100 nm after peptide binding (Figure S8A, Supporting Information), and the PDI of liposomes after peptide binding was less than 0.2 (Figure S8B, Supporting Information). Zeta potential of liposomes showed no obvious differences and were still negative after peptide binding (Figure S8C, Supporting Information). Cryo‐electron microscopy revealed that both CPQ liposomes with or without peptide library bound were spherical, with sizes close to 100 nm (Figure 3C). Each peptide library contains almost all 20 peptides and the distribution is random (Figure 3D) based on MS‐LC (Figure S9 and Table S1, Supporting Information). The median peptide distribution percentage of AH1‐Pos1, AH1‐Pos3, AH1‐Pos5 and AH1‐Pos8 were 4.3%, 3.9%, 4.45%, and 4.6%, respectively and the interquartile ranges were 2.6%–9.6%, 1.9%–6.8%, 2.8%–6.9%, and 1.4%–7.8%, respectively. All of these 4 positional libraries have over 70% of peptides express more than 2% in the peptide libraries.

The MHC‐I binding affinity of AH1 library members was predicted by NetMHC, revealing that the binding percentiles of wild‐type AH1 to H‐2L^d^, H‐2D^d^ and H‐2K^d^ are 0.5, 27, 6% respectively, indicating that AH1 is a good H‐2L^d^ binder. Any amino acid change at MHC anchor positions 2 and 9 diminished the predicted H‐2L^d^ ‐peptide binding while changes in other positions did not obviously alter the average H‐2L^d^ binding (Figure S10, Supporting Information). BALB/c mice were then immunized with CPQ and AH1 libraries or the wild‐type AH1 peptide at 1 µg total peptide dose on days 0 and 7 and blood was collected on day 14 for analysis. The wild‐type AH1 peptide did not elicit a detectable amount of AH1 tet^+^ CD8^+^ T cells but mice vaccinated with CPQ and peptide library Pos1, Pos3, Pos5 or Pos8 elicited more than 10% of CD8^+^ T cells that were Ag specific (Figure 3E). Vaccination with CPQ/Pos1, CPQ/Pos3. CPQ/Pos8 protected 100% mice from tumor challenge for at least 90 d. CPQ/Pos5 protected 2/3 mice from tumor challenge for at least 90 d (Figure 3F). Only a single mouse was protected with the wild‐type CPQ/AH1 immunization. Pos2, Pos6, Pos7, and Pos9 libraries were ineffective immunogens for tumor prevention.

**Figure 4:**
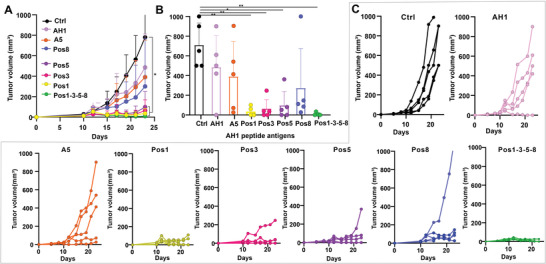
Antitumor efficacy of AH1 positional library vaccines and their mixture. BALB/c mice were inoculated with CT26 cells on day 0 and then vaccinated with CPQ and AH1 peptide libraries on days 8 and 15. A) Tumor growth of untreated mice or mice injected with indicated CPQ vaccines. Error bars show mean ± std. dev. for *n* = 5 mice. B) Tumor sizes on day 23. Error bars show mean ± std. dev. for *n* = 5 mice. C) Tumor growth of individual mice in each group with *n* = 5 mice per group. * *p* < 0.05, and ** *p* < 0.01, analyzed by (A, B) one‐way ANOVA with Turkey multiple comparisons post‐test. Asterisk in A compares tumor sizes to untreated control group on day 23.

The AH1‐A5 peptide, with alanine at position 5 is a previously characterized “e‐mimotope” of AH1; it increases the binding efficacy of pMHC complex with TCR. AH1‐A5 is also much more immunogenic as a vaccine antigen compares to AH1.^[^ ^22^ ^]^ To compare the anti‐tumor efficacy of single mimotopes as immunogens and positional libraries as immunogens, we made vaccines by admixing CPQ liposome with wild‐type peptide AH1, AH1‐A5, the positional peptide library AH1‐Pos1, AH1‐Pos3, AH1‐Pos5, AH1‐Pos8, or the pooled AH1‐Pos1‐3‐5‐8 library. The total doses of peptide Ag were kept at 1 µg per mouse. Mice were inoculated with CT26 tumor cells on day 0 then immunized with intramuscular injection on days 8 and 15. Mice vaccinated with CPQ/AH1, CPQ/AH1‐A5, and the CPQ/AH1‐Pos8 library did not show significantly slower tumor growth compared to untreated mice. However, mice vaccinated with CPQ/AH1‐Pos1, CPQ/AH1‐Pos3, CPQ/AH1‐Pos5, and CPQ/AH1‐Pos1‐3‐5‐8 delayed tumor growth significantly (**Figure** **4**A). On day 23, 4/5 of mice vaccinated with CPQ/AH1‐Pos1‐3‐5‐8 showed no evidence of tumor growth and one mouse showed an extremely small tumor which is around 2 × 3 mm (Figure 4B). However, single peptide vaccines CPQ/AH1 and CPQ/AH1‐A5 did not show significant antitumor efficacy compared to the untreated group, even though one mouse in the CPQ/AH1 was tumor free and two mice in the CPQ/AH1‐A5 group showed slower tumor growth compared to control group (Figure 4C), the average tumor growth were not delayed by these two single peptide vaccines. All mice vaccinated with other positional libraries had smaller tumor sizes than untreated mice, except one mouse from the CPQ/Pos8 group.

### 2.4 Mechanism of Enhanced Antitumor Efficacy of Positional Library Immunogens

**Figure 5:**
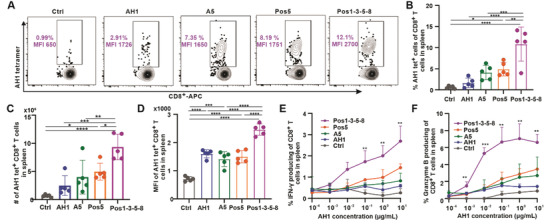
AH1 positional library immunogens induced CD8^+^ T cells with higher affinity to the AH1 tetramer and generated a greater frequency of cytokines compared to the wild‐type epitope. BALB/c mice were inoculated with CT26 cells subcutaneously on day 8 and then untreated or injected with vaccine on days 8 and 15. Splenocytes were collected on day 23. Flow cytometry gating (A) and percentage (B) of AH1 tet^+^ cells in the CD8^+^ T cell population. C) Number of AH1‐tet^+^ CD8^+^ T cells in spleen. D) Geometric medium fluorescence intensity (MFI) of AH1‐tet^+^ CD8^+^ T cells. Splenocytes were stimulated with different concentrations of the wild‐type AH1 peptide, followed by analysis of IFN‐*γ* and granzyme B expression by intracellular staining. E) Percentage of IFN‐*γ*‐producing cells and F) percentage of granzyme B‐producing cells in the CD8^+^ T cell population. Error bars in B, C, D, E, F show mean ± std. dev. for *n* = 5 mice. * *p* < 0.05, ** *p* < 0.01, *** *p* < 0.001, and **** *p* < 0.0001, analyzed by (B, C, D) one‐way ANOVA with E,F) Tukey multiple comparisons post‐test or one‐way ANOVA with Dunnett comparisons post‐test. Asterisks in panel E and F indicates statistically significant differences between indicated group and control group with indicated AH1 concentration.

To investigate why the positional library vaccine was more effective than the AH1 peptide vaccine, mice were inoculated with CT26 tumors subcutaneously on day 0 and vaccinated on days 8 and 15; splenocytes were then prepared on day 23 for tetramer analysis. With the same amount of peptide dose in each vaccine, only positional library immunogens elicited a significantly higher percentage of AH1 tet^+^ cells in the CD8^+^ T cell population compared to untreated mice (**Figure** **5**A,B). In addition, positional peptide library Pos1, Pos3, Pos5, and Pos8 combination (AH1‐Pos1‐3‐5‐8) induced an even higher percentage of AH1 tet^+^ cells in CD8^+^ T cells compared to Pos5 vaccine. Neither the AH1 wild‐type peptide nor the AH1‐A5 and e‐mimotope induced a significantly greater number of CD8^+^ T cells in the spleen compared to untreated mice; AH1‐Pos5 and AH1‐Pos1‐3‐5‐8 vaccinated mice showed significantly more Ag‐specific T cells in the spleen (Figure 5C). The mean fluorescence intensity (MFI) of tetramer staining was correlated with an increased T‐cell affinity for Ag.^[^ ^61^ ^]^ While AH1‐specific CD8^+^ T cells expanded by AH1, AH1‐A5 and AH1‐Pos5 stained with AH1 tetramer with similar intensity, the positional library combinational vaccine AH1‐P1‐3‐5‐8 stained more intensely with the AH1 tetramer compared to other AH1 immunogens (Figure 5D). We also observed that even though the percentage of AH1 tet^+^CD8^+^ T cells was not significantly different between CPQ/AH1‐vaccinated mice and untreated mice, the geometric MFIs of the induced tet^+^CD8^+^ T cells were much higher than the control group.

KLRG‐1 and IL‐7Ra markers were used to identify short‐lived effector cells (SLEC; KLRG^–^1^+^ IL‐7Ra^–^). SLEC are terminally differentiated T cells with immediate cytolytic effector function and increased expression of the transcription factor T‐bet.^[^ ^62^ ^]^ While both positional library vaccines (CPQ/AH1‐Pos5 and CPQ/AH1‐Pos1‐3‐5‐8) showed an increase in KLRG‐1^+^IL‐7Ra^+^ cells in CD8^+^ T cells, the positional library vaccine CPQ/Pos1‐3‐5‐8 showed the highest percentage of KLRG‐1^+^IL‐7Ra^+^ cells among all the groups (Figure S11A,B, Supporting Information). Mice vaccinated with the e‐mimotope AH1‐A5, the positional library vaccine AH1‐Pos5 and the pooled libraries AH1‐Pos1‐3‐5‐8 displayed an increase in SLEC‐expressing cells (Figure S11C, Supporting Information). In contrast, the CPQ/AH1 vaccinated mice had no increase in SLEC‐expressing cells compared to the untreated group. For CPQ/Pos1‐3‐5‐8 vaccinated mice, a similar result could be observed in blood (Figure S11D–F, Supporting Information).

Next, we tested cytokine production by CD8^+^ T cells in response to vaccination. We stimulated splenocytes from vaccinated mice with increasing wild‐type AH1 peptide concentrations and measured intracellular IFN‐*γ* and granzyme B production. In splenocytes from CPQ/AH1‐Pos1‐3‐5‐8 vaccinated mice, there were the highest percentages of IFN‐*γ* and granzyme B producing CD8^+^ T cells, which correlated with increasing concentrations of AH1 stimuli (Figure S12, Supporting Information, Figure 5E,F). In addition, AH1‐Pos5 group showed slightly higher percentages of IFN‐*γ* and granzyme B producing cells in the CD8^+^ T cell population compared to the AH1‐A5 e‐mimotope. In contrast, the percentage of CD8^+^ T cells that produced IFN‐*γ* and granzyme B were much lower in response to CPQ/AH1 vaccination even at the highest re‐stimulation peptide concentration. This result correlated with the extent of AH1 tet^+^ CD8^+^ T cells resulting from vaccination with the AH1 positional library vaccine compared to the other platforms. However, when IFN‐*γ* and granzyme B production were plotted as a percentage of AH1 tet^+^ cells, with a higher peptide concentration during restimulation a higher frequency of AH1 specific T cells producing IFN‐*γ* and granzyme B were observed. CPQ/AH1‐Pos1‐3‐5‐8 group had a much higher frequency of AH1 specific T cells produced IFN‐*γ* (Figure S13A, Supporting Information) and granzyme B (Figure S13B, Supporting Information) compared to all other groups. This indicates that a higher frequency of AH1‐specific CD8^+^ T cells elicited by the AH1‐Pos1‐3‐5‐8 library vaccine is reactive with the wild‐type AH1 peptide compared to T cells elicited by AH1‐A5 or AH1 vaccination.

Considering the potential for the positional library immunogens to induce self‐reactive CD8^+^ T cells, safety studies were carried out. Tumor‐bearing BALB/c mice were immunized on days 12 and 19 with CPQ/Pos1‐3‐5‐8. Mice exhibited normal weight gain (Figure S14A, Supporting Information). On day 26, organs were removed and no obvious changes in organ weights were observed (Figure S14B, Supporting Information). A serum chemistry panel (Figure S14C, Supporting Information) revealed that all parameters of vaccinated mice either showed no difference compared to untreated mice or were in the normal range for healthy mice.

### 2.5 Vaccination with a Positional Peptide Library Nanoparticle Formulation Elicits Epitope‐Specific CD8^+^ T Cells with Higher TCR Clonal Diversity

**Figure 6:**
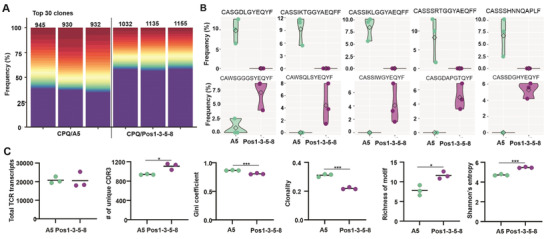
Positional peptide library immunogens induce expansion of diverse epitope‐specific CD8^+^ T cell repertoires. Mice were vaccinated with CPQ/A5 or CPQ/Pos1‐3‐5‐8 on days 0 and 7, and tet^+^CD8^+^ T cells were sorted from splenocytes on day 14, followed by DNA extraction and TCR sequencing. A) The 30 most abundant amino acid clonotype frequencies from each sample are multicolored while the remaining cumulative clone frequency is shown in purple. The total number of unique CDR3 clonotypes observed in each sample is noted. B) The top 5 most significantly differentially abundant tet^+^CD8^+^ TCR clones enriched in CPQ/A5 relative to CPQ/Pos‐1‐3‐5‐8 (top) and conversely enriched in CPQ/Pos1‐3‐5‐8 relative to CPQ/A5 (bottom). CDR3 sequences are indicated. C) Repertoire metrics, showing the number of total TCR transcripts detected in each sample, number of unique CDR3 sequences observed, Gini coefficient, clonality, richness of motif and Shannon entropy of the TCR of tet^+^CD8^+^ T cells from CPQ/A5 and CPQ/Pos1‐3‐5‐8 vaccinated mice. The experiment was performed with *n* = 3 independent mice per group and the line shows the mean. * *p* < 0.05, ** *p* < 0.01, and *** *p* < 0.001, analyzed by unpaired student *t* test.

To better understand the mechanism of the observed enhanced antitumor efficacy of the positional library vaccine compared to the single e‐mimotope vaccine, mice were vaccinated with either CPQ/A5 or CPQ/Pos1‐3‐5‐8 on days 0 and day 7. On day 14, AH1‐tet^+^CD8^+^ T cells were collected from splenocytes and DNA was extracted for TCR sequencing. Lorenz curves showed that TCR clonotypes were not equally distributed within the AH1‐tet^+^CD8^+^ T cell repertoires induced by either vaccine (Figure S15A, Supporting Information), and indeed both immunization methods generated clonally expanded TCR clonotypes (Figure S15B, Supporting Information). Consistent with these findings, the clonality of AH1‐specific CD8^+^ T cells induced by either CPQ/Pos1‐3‐5‐8 or CPQ/A5 vaccination was high. The 30 most frequent T cell clones accounted for more than 60% of the TCR sequences observed in the CPQ/A5 vaccinated group and ≈40% of the CPQ/Pos1‐3‐5‐8 group (multi‐colored portion of graphs in **Figure** **6**A). Reciprocally, ≈40% of AH1‐specific CD8^+^ T cells induced by CPQ/A5 and ∼60% from CPQ/Pos1‐3‐5‐8 group correspond to less frequent clones (Purple).

However, differential abundance analysis of TCR‐CDR3 sequences (Figure S15C, Supporting Information, Figure 6B) indicated that the individual TCR clones induced by CPQ/A5 and CPQ/Pos1‐3‐5‐8 were different, which could contribute to differential anti‐tumor efficacy. While the total number of sequenced TCR transcripts was similar between the two groups, mice immunized with CPQ/Pos1‐3‐5‐8 had substantially higher numbers of unique CDR3 regions with reduced measures of Gini coefficient clonality, increased richness of motif and Shannon's entropy, reflecting a more diverse T cell‐repertoire within the clonal tumor‐specific T‐cell population, compared to mice immunized with CPQ/A5 (Figure 6C). Similarity assessment of TCR repertoires between samples showed that TCR heterogeneity existed within the AH1‐tet^+^ CD8^+^ T cells between individual mice, which was especially prominent in mice from different vaccine group (Figure S15D, Supporting Information).

### 2.6 Synergistic Effects of Vaccine and Immune Checkpoint Blockade

**Figure 7:**
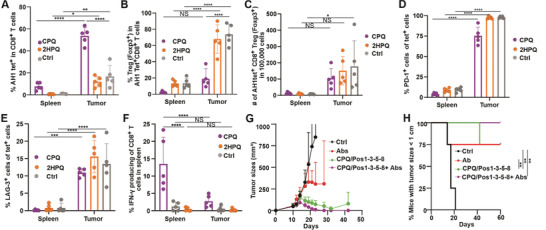
Synergistic effects of CPQ/Pos1‐3‐5‐8 vaccine and PD‐1 and CTLA‐4 monoclonal antibodies combination therapy. BALB/c mice were inoculated with CT26 cells subcutaneously on day 0 and immunized 12 and 19 d later. Spleen and tumor tissues were collected 26 days post‐tumor inoculation for CD8^+^ T cell analysis. A) Percentage of AH1 tet^+^ cells within the CD8^+^ T cell population. B) Percentage of Treg (Foxp3^+^) cells within the tet^+^CD8^+^ T cells. C) Number of tet^+^CD8^+^ Treg in 100 000 cells. Percentage of PD‐1^+^ (D), LAG‐3^+^ (E) cells within the tet^+^CD8^+^ T cells. F) IFN‐*γ* ^+^ cells within tet^+^CD8^+^ T cells after 10 µg mL^–1^ Ag stimulation in vitro. Error bars in A, B, C, D, E, F show mean ± std. dev. for *n* = 5 mice per group. BALB/c mice were inoculated with CT26 cells on day 0 and either remained untreated or vaccinated with CPQ/Pos1‐3‐5‐8 on days 10 and 17 or vaccinated with CTLA‐4 and PD‐1 antibodies on day 12, 14, 19, 21 (Abs) or vaccinated with vaccine on day 10 and 17 and CTLA‐4 and PD‐1 antibodies on day 12, 14, 19, 21 (CPQ/Pos1‐3‐5‐8+Abs). G) Tumor growth of mice. H) Mice with tumor sizes smaller than 1 cm. Error bars in G, H show mean ± std. dev. for *n* = 4 mice per group. * *p* < 0.05, ** *p* < 0.01, *** *p* < 0.001 and **** *p* < 0.0001, analyzed by two‐way ANOVA (A, B, C, D, E, F) with Bonferroni multiple comparisons post‐test or long rank test (H).

Next, we studied the Ag‐specific T cells in the tumor microenvironment (TME). BALB/c mice were inoculated with tumors on day 0 and then vaccinated on days 12 and 19. Splenocytes and tumor‐infiltrating lymphocytes (TILs) were prepared on day 26. With CPQ/Pos1‐3‐5‐8 vaccination, ≈10% and 50% of CD8^+^ T cells were Ag‐specific in the spleen and tumor, respectively (Figure S16, Supporting Information, **Figure** **7**A). With CPQ vaccination, the number of T_EM_ AH1‐tet^+^ cells increased dramatically and T_CM_ tet^+^ cells and stem memory T cells (T_SCM_) tet^+^ cells increased slightly in the spleen but not in the tumor and blood (Figure S17, Supporting Information). Among these AH1‐tet^+^ CD8^+^T cells, ≈2% and 20% were T regulatory (Treg) cells in the spleen and tumor, respectively (Figure 7B). There was no increase in the number of Treg cells with CPQ vaccination compared to 2HPQ vaccination or with the untreated group (Figure 7C), but the number of Ag‐specific Treg cell increased in the tumor compared to the spleen.

We also assessed the expression of programmed cell death protein 1 (PD‐1) and lymphocyte‐activation gene 3 (LAG‐3) to assess T‐cell exhaustion of the Ag‐specific T cells in the TME. In the TME, a much higher percentage of AH1‐specific CD8^+^ T cells expressed PD‐1 (Figure 7D) and LAG‐3 (Figure 7E) compared to the spleen. Splenocytes and TILs were stimulated with Ag in vitro, and ≈15% and 3% of CD8^+^ T cells produced IFN‐*γ* in the spleen and tumor, respectively, with CPQ/Pos1‐3‐5‐8 vaccination. 2HPQ/Pos1‐3‐5‐8 vaccinated mice showed no difference compared to the untreated group and both groups had much lower percentages of IFN‐*γ*‐producing cells in the CD8^+^ T cell population compared to the CPQ group (Figure 7F).

Next, we combined vaccine and immune checkpoint inhibitor (ICI) antibodies to enhance antitumor responses. BALB/c mice were implanted with CT26 tumor cells on day 0 and then vaccinated on day 10 when tumor sizes were ≈100 mm^3^ followed by boosting 7 d after. Since the ICIs combined with the vaccine could potentially restore the antitumor function of T cells,^[^ ^63^ ^]^ in our study, the ICIs were administered 2 and 4 d after CPQ/Pos1‐3‐5‐8 vaccination. We observed that tumor size reached 200 mm^3^ and started to shrink on day 14. Mice treated with vaccine and ICI eradicated tumors completely. Mice injected only with vaccine inhibited tumor growth, but after day 30, tumor regrowth occurred (Figure 7G). The ICI only group, vaccine only group, and the combination therapy group had higher percentages of mice with tumor sizes smaller 1 cm compared to the control group (Figure 7H).

## 3 Discussion

A current challenge in the clinical translation of cancer peptide vaccines is the selection of tumor‐associated and tumor‐specific epitopes that give rise to CD8^+^ T cell responses to kill tumor cells. Current approaches typically involve the multiplexing of antigens into a single vaccine, typically, 6‐20 peptides, to improve the odds of inducing functional T‐cell responses.^[^ ^64^, ^65^, ^66^, ^67^, ^68^, ^69^ ^]^ Direct targeting of CD8^+^ T‐cell neoepitopes has been particularly challenging, which has necessitated the use of longer immunogens ranging from 20 to up to hundreds of amino acids in length, which result in anti‐tumor effects driven by MHC‐II, not MHC‐I responses.^[^ ^70^ ^]^ The data from the present study suggest that positional library immunogens could potentially be useful in the context of targeting MHC‐I epitopes judged to be important and expressed on target cells.

The position‐scanning peptide library vaccine approach depends on a known T‐cell epitope. The classical way to find T‐cell epitopes involves the use of an isolated T‐cell clone to recognize T‐cell epitopes that were presented on the surface of target cells.^[^ ^71^, ^72^ ^]^ Progress in T‐cell epitope discovery using genomic and proteomic approaches facilitates the process. Besides this, T‐cell epitopes have been discovered by MHC display affinity^[^ ^40^ ^]^ and combinatorial tetramer staining and mass cytometry analysis.^[^ ^73^ ^]^ In recent years, high‐throughput T‐cell antigen profiling technologies such as identifying CD8^+^ T‐cell epitopes from peptide‐coding library holds potential to make T‐cell epitope identification even more efficient.^[^ ^74^ ^]^ Taken together, considering that technological approaches will likely facilitate the identification of CTL target epitopes, approaches to enhance their immunogenicity such as the one presented here could be useful.

TCR sequencing analysis demonstrated that the peptide library vaccine approach, which induced stronger anti‐tumor efficacy compared to A5 vaccination, also produced more diverse Ag‐specific T‐cell repertoires. Since low‐affinity T‐cell repertoires have been shown to be the main effectors in the immune response and interact with self‐tumor antigens,^[^ ^37^, ^39^ ^]^ we hypothesized that the position‐scanning peptide library immunogen was effective in activating a low‐affinity T‐cell repertoire that was reactive with the wild‐type epitope.

Although the current study was limited to mice, the concept of using positional peptide library immunogens could be broadly applicable to other species such as humans, where MHC‐I epitopes are also ≈9 amino acids in length. We would hypothesize that in other species, T‐cell repertoires induced by peptide library vaccination would also be more diverse than single peptide vaccination with wild‐type or e‐mimotope vaccines. Further testing of this notion could first be carried out in humanized mouse models. With respect to potential for clinical translation, CoPoP is currently undergoing human clinical trial testing for a SARS‐CoV‐2 vaccine (clinicaltrials.gov # NCT04783311), so there is now precedence to use this particle immunogen approach in humans.

The regulatory requirements for using a peptide library in terms of chemical characterization are also unclear and could pose a practical challenge, as the library represents a pool of many peptides. Other challenges remain in the development of positional library immunogens. As currently synthesized by solid‐phase peptide synthesis, we observed a broad distribution of individual peptide members within the positional libraries. It is not clear whether functional but under‐represented peptides would be sufficiently present in the vaccine to have an impact on immunogenicity. However, the ease of synthesis of a single peptide library likely outweighs the accuracy of an alternative that would involve the synthesis of all twenty peptides of the library and pooling them. Another limitation is which positions should be rendered into libraries. While none of the effective positional libraries we found were localized at H‐2L^d^ anchor residues (Pos2 and Pos9), further work would be needed to confirm those positions could be omitted from screening or if certain sites are consistently better than others. Pos5 was effective in both the two peptide systems tested, but more work within H‐2L^d^ and other MHC haplotypes is needed for further understanding patterns in the library immunogens.

## 4 Conclusion

We propose a new vaccination strategy of using a short peptide positional library that substitutes one position of the peptide sequence with a mixture of all 20 amino acids, while leaving the remainder of the peptide sequence the same as wild‐type. These libraries are presented on the surface of highly immunogenic liposomes for immunization. We found that an Env_37‐44_ positional library Pos5 dramatically increased the frequency of Ag‐specific CD8^+^ T cells. However, a limited number of these elicited T cells could cross react with wild‐type peptide and secrete cytokines after wild‐type peptide stimulation. The same strategy was applied to the AH1 peptide. We identified AH1 positional libraries AH1‐Pos1, AH1‐Pos3, AH1‐Pos5, and AH1‐Pos8 were able to induce a high level of Ag‐specific T cells that inhibited CT26 tumor growth. These libraries and the library mixture AH1‐Pos1‐3‐5‐8 exhibited higher antitumor efficacy compared to previously established e‐mimotope AH1‐A5 and the wild‐type peptide AH1. Positional libraries induced T cells with the highest affinity to the AH1 tetramer and increased the frequency of T cells to produce cytokines in the activation state. A more diverse tumor‐specific TCR repertoire was induced by AH1‐Pos1‐3‐5‐8 compared to A5 vaccination. In addition, the positional library vaccine is also synergistic with ICIs targeting CTLA‐4 and PD‐1.

## 5 Experimental Section

### Materials

Peptides and peptide libraries were synthesized by GenScript and were used as is without further assessment of peptide concentration. CoPoP was produced as previously described.^[^ ^75^ ^]^ The following lipids were used: Dioleoylphosphatidylcholine (DOPC) (Corden; catalog number: LP‐R4‐070), cholesterol (PhytoChol; Wilshire Technologies), synthetic Monophosphoryl Hexa‐acyl Lipid A, 3‐Deacyl PHAD‐3D6A (Avanti; catalog number: 699855), and QS‐21 (Desert King; catalog number: NC0949192). It is noted that PHAD‐3D6A was the only MPLA used in this study, and where PHAD is mentioned, this refers to PHAD‐3D6A. The following antibodies were obtained from BioLegend, CD45‐AF700 antibody (0.5 mg mL^–1^, catalog number: 103127), APC‐CD8a antibody (0.2 mg mL^–1^, catalog number: 100712), FITC‐CD4 antibody (0.5 mg mL^–1^, catalog number: 100405), PE‐Cy7‐CD4 antibody (0.2 mg mL^–1^, catalog number: 100527), APC‐Cy7‐CD44 (0.2 mg mL^–1^, catalog number: 103027), PE/Cy7‐CD62L antibody (0.2 mg mL^–1^, catalog number: 104417), FITC‐Sca‐1 (0.2 mg mL^–1^, catalog number: 108105), Foxp3‐AF488 (0.5 mg mL^–1^, catalog number: 126405), PerCP‐Cy5.5‐PD‐1 (0.2 mg mL^–1^, catalog number: 109119), PE‐Cy7‐LAG‐3 (0.2 mg mL^–1^, catalog number: 125225), Pacific Blue‐IFN‐*γ* (0.5 mg mL^–1^, catalog number: 505818), PE‐TNF‐*α* (catalog number: 506305), PE‐Cy7‐Granzyme B (catalog number: 372213), APC‐Cy7‐KLRG1 (0.2 mg mL^–1^, catalog number: 138425), PE‐Cy7‐IL‐7R*α* (0.2 mg mL^–1^, catalog number: 135013). Other reagents included: Brefeldin A/Golgiplug (BD; catalog number: 555029), Live/Dead dye (Invitrogen; catalog number: L34857), Fc‐block (BD; catalog number: 553142). Fixation/permeabilization kit (BD; catalog number: 554714), True‐Nuclear transcpription factor buffer set (BioLegend, catalog number: 424401), Cell lysis buffer (BioVision; catalog number: 5830). Collagenase Type I (Gibco; catalog number: 17018–029), DNase I (Roche Diagnostics; catalog number: 04536282001). Anti‐CTLA‐4 (Clone: 9H10, BioXCell, catalog number: BP0131), Anti‐PD‐1(Clone: RMP1‐14, BioXCell, catalog number: BP0146). ELISpot (MABTECH, catalog number: 3321‐4ATP‐2).

### Methods: Vaccine Preparation and Characterization

CPQ liposomes were prepared by ethanol injection and lipid extrusion, as reported previously.^[^ ^53^ ^]^ Additional details are provided in the Supporting Information. Ethanol was removed from prepared liposomes by dialyzing in phosphate‐buffered saline (PBS) at 4 °C, then liposomes were passed through a 0.2 µm sterile filter. For CPQ and 2HPQ preparation, QS‐21 (1 mg mL^–1^) was added to liposomes overnight at 4 °C for a [DOPC: Cholesterol: CoPoP/PoP: PHAD: QS‐21] mass ratio of [20:5:1:0.4:0.4]. The final liposome concentration was adjusted to 320 µg mL^–1^ CoPoP or 2HPoP by dilution in PBS. To prepare the CPQ and 2HPQ vaccine, liposome and peptides or positional peptide libraries were incubated at a mass ratio of 4:1 for 1 h at room temperature. Liposomes were then diluted to achieve an injecting dose of peptides and peptide libraries of 1 µg antigen per mouse in 50 µL PBS.

To characterize the binding of peptides to liposomes, peptides were incubated with liposomes or PBS for an hour at room temperature and then subjected to microcentrifugal filtration tube with a 100 kDa cutoff (PALL; catalog number: 29300) to separate free peptide from liposomes. To determine free peptide concentration in the filtrate, micro BCA (Thermo; catalog number: 23235) assays were used. Dynamic light scattering with a NanoBrook 90 plus PALS instrument was used to measure sizes and polydispersity index (PDI) of dilute samples in PBS and zeta potential in water.

LC‐MS was used to characterize the peptide distribution in positional peptide libraries. Separation of the positional peptide libraries (4 mg mL^–1^ in PBS) was performed using a Shimadzu Ultra‐High Performance Liquid Chromatography (UHPLC) system (Nexera XR; Shimadzu Scientific Instruments, Inc., Columbia MD, USA) on a reverse‐phase SunFire C8 column (2.1 × 50 mm, 100 Å; 5 µm; Waters (Milford, MA, USA)). The mobile phase was composed of A (H_2_O + 0.1% trifluoroacetic acid (TFA)) and B (acetonitrile (ACN)). The flow rate was 0.4 mL min^–1^ and the column oven was kept at ambient temperature. 5 µL of peptide libraries Env_37‐44_‐Pos5, AH1‐Pos1 and AH1‐Pos3 or 20 µL of peptide libraries AH1‐Pos5 and AH1‐Pos8 were injected via the autosampler. The solvent composition was held at 5% B for 5 min. A mobile phase gradient from 5% to 40% B was then applied from 5 to 65 min, followed by a 5 min isocratic wash at 95% B and finally a 5 min re‐equilibration step (5% B). This method was used as a method for generic peptide separation, therefore peak overlap was expected. Overlapping MS signals are easily compensated by the fast scan speed of the triple quadrupole instrument.

An LC‐MS‐8045 triple quadrupole mass spectrometer (Shimadzu Scientific Instruments, Inc., Columbia, MD, USA) was used. Electrospray ionization (ESI) in positive mode was employed and the mass spectrometer was operated using a Q3 scan event (400–2000 m z^–1^). The operation parameters were as follows: interface temperature at 300 °C; interface voltage at 4 kV; desolvation line temperature at 250 °C; heat block temperature at 400 °C; heat gas flow, nitrogen at 10 L min^–1^; and drying gas flow, nitrogen at 10 L min^–1^; nebulizing gas flow at 2 L min^–1^. LC‐MS data processing was performed using Shimadzu LabSolutions software (Version 5.96). The [M+2H]^2+^ ion—the most intense ion in the multiply‐charged ion envelope for each peptide in the library—was extracted from the total ion chromatogram and the corresponding peak areas were calculated. Amino acid distribution of peptide libraries was determined by the peak area of indicated peak to the peak area of all peaks per peptide library.

For cryo‐electron microscopy, to analyze the morphology of CPQ liposomes before and after binding of positional peptide libraries, approximately 3.6 µL of each sample was applied to the holey carbon grids and manually blotted using the Vitrobot blotting paper (Standard Vitrobot Filter Paper, Ø55/20 mm, Grade 595). Right after blotting, a new drop of the sample was applied to the EM grid and blotted again using the standard routine with the two blotting pads in the Vitrobot Mark IV (Thermo Fisher Scientific) for 3 s and a blot force +1. The grid was then immediately plunged into liquid ethane. The Vitrobot was set at 25 °C and 100% relative humidity. For all samples, c‐flat grids (C‐Flat 2/2‐3Cu‐T) were used, which were washed with chloroform for 2 h and treated with glow discharge in air at 5 mA for 15 s right before the sample was applied for vitrification. Samples were imaged in a Tecnai F20 electron microscope operated at 200 kV using a side‐entry Gatan 626 single tilt cryo‐holder. Images were collected in a TVIPS XF416 CMOS camera at a magnification of 62000 ×, which produced images with a calibrated pixel size of 1.716 Å. Images were collected with a total dose of  ≈51 e^–^ Å using a defocus ranging from −1.75 to − 2.50 µm.

### Cell Studies

CT26 cells were obtained from American Type Culture Collection (ATCC) and cultured in Roswell Park Memorial Institute (RPMI) 1640 with 10% fetal bovine serum (FBS) and 1% penicillin/streptomycin (pen/strep). For splenocyte studies, freshly isolated spleens were dissociated and filtered through a 70 µm cell strainer. The plunger from a sterile 3 mL syringe was used to dissociate tissue through the strainer, and 5 mL of cold PBS was used to wash cells into a 50 mL tube. Cells were centrifuged at 500 × *g* for 5 min, and the supernatants were discarded. Red blood cells were lysed with a 5 mL red blood cell lysis (RBC) buffer for 5 min, then 35 mL PBS was added to the tube. Cells were centrifuged again, and the cell pellets were collected for further use. For tumor‐infiltrating lymphocyte (TIL) studies, tumor was isolated and cut to 1–2 mm pieces then digested with collagenase Type I (2 mg mL^–1^) and DNase I (100 µg mL^–1^) for an hour in the cell culture incubator. Then the plunger from a sterile 3 mL syringe was used to dissociate tumor tissue through the 70 µm strainer, cells were collected and washed for further experiment. Peripheral blood mononuclear cells (PBMCs) were prepared by lysing 100 µL of whole blood with 2 mL of RBC lysis buffer and washed twice for further use. Splenocytes, TILs, and PBMCs were cultured in RPMI 1640 supplemented with 10% FBS, 1% pen/strep, glutamine (2 × 10^−3^ m), sodium pyruvate (1 × 10^−3^ m), 1x diluted nonessential amino acids solution, and *β*‐mercapethanol (50 × 10^−6^ m). Cells were cultured in 5% CO_2_/95% air at 37 °C in a humidified chamber.

### Murine Studies

Murine studies were performed according to protocols approved by the University at Buffalo IACUC (approval No. BME13028Y). 5‐6‐week‐old female BALB/c mice (Charles River Laboratory) were intramuscularly immunized with 50 µL vaccine on the right quadriceps. For the prophylactic vaccine tumor model, mice were vaccinated on days 0 and 7 and challenged on day 14. For the therapeutic vaccine tumor model, mice were inoculated with 3 × 10^4^ tumor cells subcutaneously on day 0, and then vaccinated with the indicated vaccine on days 8 and 15. For TME experiments, mice were inoculated with 3 × 10^4^ tumor cells subcutaneously on day 0, and then vaccinated with the indicated vaccine on days 12 and 19, tumors and spleens were harvested on day 26. For vaccine and PD‐1 and CTLA‐4 combinational therapy, mice were inoculated with 3 × 10^4^ tumor cells subcutaneously on day 0, and then vaccinated intramuscularly on days 10 and 17. PD‐1 (100 µg per mouse) and CTLA‐4 (100 µg per mouse) were vaccinated intraperitoneally on days 12, 14, 19, and 21. Tumor growth was monitored three times a week and tumor sizes were calculated by the equation: Tumor volume = length × width^2^/2. Animals were euthanized when the tumor sizes reached 1 cm in diameter or when animals developed an ulceration.

### Antibody Staining

For tetramer staining, immunized mice were analyzed for the percentages of tet^+^ cells of CD8^+^ T cells by a tetramer staining assay. H‐2L^d^‐restricted Env_37‐44_ (SPHQVFNL) and AH1 (SPSYVYHQF) peptides were complexed with MHC‐I (H‐2L^d^) and conjugated with PE (the NIH Tetramer core facility). PBMC from 100 µL of blood or 1 × 10^6^ splenocytes or 5 × 10^6^ TIL were incubated with the tetramer (100 × diluted) for 1 h at 4 ℃. For T‐cell phenotyping, antibody mixture of Fc‐block (100 × diluted), CD45 (Clone: 30‐F11; 200 × diluted), CD8a (Clone: 53‐6.7; 200 × diluted), CD4 (Clone: RM4‐5; 200 × diluted), CD44 (Clone: IM7; 200 × diluted) and CD62L (Clone: MEL‐14; 200 × diluted), Sca‐1 (D7, 200 × diluted) or antibody mixture of Fc‐block (Clone: 2.4G2; 100 × diluted), CD8a (Clone: 53‐6.7; 200 × diluted), CD4 (Clone: GK1.5; 200 × diluted), KLRG‐1 (Clone: 2F1; 200 × diluted) and IL‐7R*α* (Clone: A7R34; 200 × diluted) were added to cells. For T cell exhaustion study, antibody mixture of Fc‐block (Clone: 2.4G2; 100 × diluted), CD8a (Clone: 53‐6.7;200 × diluted), CD4 (Clone: GK1.5; 200 × diluted), PD‐1 (Clone: RMP1‐30; 200 × diluted) and Lag‐3 (Clone: C9B7W; 200 × diluted) were added to cells. Cells were incubated with these antibodies for 30 min at 4 °C, then, washed twice for flow cytometry analysis. Flow cytometry studies were carried out using a BD LSRFortessa X‐20 cytometer. Flowjo (version 10) software was used for data analysis.

### For Intracellular Staining

PBMCs from 100 µL blood, 1 × 10^6^ splenocytes or 5 × 10^6^ TIL in 100 µL cell culture medium were seeded in a flat bottom 96‐wells plate and stimulated with antigen for 15–18 h in the cell culture incubator. Then GolgiPlug (Brefeldin A) was added to the wells at the recommended final dilution of 1000 × the stock concentration for another 5 h. Cells were transferred to a 96‐well round bottom plate and centrifuged at 1350 rpm. Cell pellets were washed twice and stained with tetramer for 1 h at 4 ℃ then stained with live/dead fixable dye (500 × diluted), Fc‐block (Clone: 2.4G2; 100 × diluted) and the following antibodies against CD45 (Clone: 30‐F11; 200 × diluted), CD8 (Clone: 53‐6.7; 200 × diluted) and CD4 (Clone: GK1.5; 200× diluted) for 25 min at 4 °C. Cells were fixed, permeabilized according to the manufacture's instruction. Cells were further stained with antibodies against IFN‐*γ* (Clone: XMG1.2; 200 × diluted), TNF‐*α* (Clone: MP6‐XT22; 200 × diluted) or Granzyme B (Clone: QA16A02; 200 × diluted) for 30 min at 4 ℃, then washed for flow cytometry. For Treg staining, after tetramer and cell surface staining, cells were fixed and premetallized according to the manufacture's instruction. Cells were further stained with antibody against Foxp3 (Clone: MF14; 200 × diluted).

For ELISpot assays, 3 × 10^5^ splenocytes or TIL were seeded to ELISpot plates and 10 µg mL^–1^ ENV_37‐44_ peptide was added to each well. Cells were cultured in 5% CO_2_/95% air at 37 °C in a humidified chamber for 24 h. Then the detection of spots was performed according to manufacturer's instruction. Images were taken by Echo REBEL microscopy with 4x objective. A number of spots were counted by image J.

For cytotoxic T lymphocyte (CTL) cytotoxicity assay, isolated splenocytes were cultured in the cell culture medium and stimulated by mouse IL‐2 (Pepro tech; catalog: 212‐12; 10 IU mL^–1^) and Ags (10 µg mL^–1^) for 5 d to use as the effector cells. 5000 CT26 cells, as target cells, were seeded in a 96‐well plate and pulsed with Ags (10 µg mL^–1^) or without Ag for an hour at 37 ℃. Then splenocytes were added to the plate at different E:T ratios for 5 h. The cytotoxicity of splenocytes on CT26 cells was assessed by lactate dehydrogenase (LDH) release using Non‐Radioactive Cytotoxicity Assay Kit (Promega; catalog: G1780) according to manufacturer instructions.

### Acute Toxicity Studies

BALB/c mice were inoculated with tumor on day 0, then either untreated or injected with CPQ/Pos1‐3‐5‐8 on days 12 and 19 intramuscularly, with doses of 1 µg AH1Pos1‐3‐5‐8 peptide, 4 µg CoPoP, 1.6 µg PHAD and 1.6 µg QS‐21 per mouse. On day 26, serum was collected for a comprehensive chemistry panel (IDEXX BioAnalytics). Organs were collected, washed, and weighed.

### TCR Sequencing

Mice were vaccinated with CPQ/A5 or CPQ/Pos1‐3‐5‐8 on day 0 and day 7, then splenocytes were prepared on day 14 and stained with AH1 tetramer and CD8 antibody for 1 h at 4 ℃. Cells were sorted then DNA from flow‐isolated murine splenic tet^+^ CD8^+^ T cells was extracted using QIAamp DNA Micro Kit (QIAGEN). DNA was quantified using Nanodrop one C (ThermoFisher). Amplification and sequencing of TCR*β* CDR3 regions were performed using ImmunoSEQ immune profiling system at the survey level (Adaptive Biotechnologies). T‐cell repertoires, comprising all detected CDR3 sequences with annotated V and J gene segment identifications were downloaded directly to the ImmunoSEQ Analyzer from Adaptive Biotechnologies. TCR repertoire analysis was performed as described.^[^ ^50^, ^76^ ^]^ Briefly, metrics of the complete TCR repertoire in each sample, including the number of productive rearrangements, productive clonality, and clonal frequencies were determined using the ImmunoSEQ Analyzer software and confirmed using the LymphoSeq package.^[^ ^77^ ^]^ On average, 20644.5 TCR templates were detected from tumor samples (range 17953‐25311), representing an average of 1021.5 unique clonotypes (range 930‐1155). Repertoires were analyzed using the LymphoSeq package and custom scripts in the R statistical software environment. The level of similarity between the different TCR repertoires was measured using the Morisita‐Horn Index,^[^ ^78^ ^]^ using the vegan package. This unitless index ranging from 0 to 1, considers the number of shared sequences between 2 repertoires as well as the contribution of those shared sequences to each repertoire. Differential clone frequencies between groups were determined using the DESeq2.^[^ ^79^ ^]^ TCR repertoires were visualized as weighted dendrograms using ImmunoMap.^[^ ^80^ ^]^ All productive sequences were considered for analysis. Sequence distances were calculated based on sequence alignments scores using a PAM10 scoring matrix and gap penalty of 30. Circles are overlaid at the end of the branches corresponding to the CDR3 sequences with diameters proportional to the frequency of the sequences observed in the samples.

### Statistical Analysis

Data were analyzed with Prism 9 (GraphPad Software) using the tests described in the figure captions. *P* values less than 0.05 were considered statistically significant. Values are generally reported as mean ± S.D. with the indicated sample size unless otherwise indicated.

## Conflict of Interest

J.F.L. and W‐C.H. holds interest in POP Biotechnologies. Other authors declare no competing interests.

## Supporting information

Supporting Information

## Data Availability

Data are available on request from the authors.
