# Supplementary material for: Position‐Scanning Peptide Libraries as Particle Immunogens for Improving CD8+ T‐Cell Responses
Source: Adv Sci (Weinh). 2021 Oct 30;8(24):2103023. doi: 10.1002/advs.202103023 (PMC8693074; doi:10.1002/advs.202103023)
Supplement: Supplementary file 1 — Supporting Information [file ADVS-8-2103023-s001.pdf]

© 2021 Wiley-VCH GmbH

ADVANCED SCIENCE
Open Access

Supporting Information

for *Adv. Sci.*, DOI: 10.1002/advs.202103023

Position-Scanning Peptide Libraries as Particle Immunogens for Improving CD8+ T Cell Responses

Xuedan He[1], Shiqi Zhou[1], Breandan Quinn[1], Weichiao Huang[1], Dushyant Jahagirdar[2], Michael Vega[3], Joaquin Ortega[2], Mark D. Long[4], Fumito Ito[5], Scott I. Abrams[5], Jonathan F. Lovell[1]*

Supporting Information: Position-Scanning Peptide Libraries as Particle Immunogens for Improving CD8$^+$ T Cell Responses

*Xuedan He[1], Shiqi Zhou[1], Breandan Quinn[1], Weichiao Huang[1], Dushyant Jahagirdar[2], Michael Vega[3], Joaquin Ortega[2], Mark D. Long[4], Fumito Ito[5], Scott I. Abrams[5], Jonathan F. Lovell[1]**

[1.] University at Buffalo, State University of New York, Buffalo, NY, 14260, USA
[2.] Department of Anatomy and Cell Biology, McGill University Montreal, Quebec, Canada
[3] Division of Research and Innovation Partnerships, Northern Illinois University, DeKalb, IL 60115, USA
[4] Department of Cancer Genetics and Genomics, Roswell Park Comprehensive Cancer Center (RPCCC), Buffalo, NY 14263, USA
[5.] Department of Immunology, Roswell Park Comprehensive Cancer Center, Buffalo, NY, 14263, USA

*E-mail: jflovell@buffalo.edu

Methods

Liposome preparation: First, CoPoP/PHAD liposomes were prepared with a [DOPC: Chol: PHAD: CoPoP] mass ratio of 20:5:1:0.4. Dry lipids were dissolved in 1 mL ethanol at 50 ˚C for 10 min and samples were sonicated for 5 seconds to break up large particles. 4 mL of PBS was added to the liposome solution and incubated for another 10 min. The liposome mixture was subjected to nitrogen pressurized liposome extrusion (Northern Lipids), which was preheated to 50 ˚C. The pressure of the extruder was around 200 PSI and the membrane stack consisted of 200, 100 and 80 nm filters. Solutions were extruded for 15 times then dialyzed in PBS for at least 4-6 hr at 4 °C two times to remove ethanol. To prepare CoPoP/PHAD/QS21 (CPQ) liposomes, QS21 was added to the CoPoP/PHAD (CP) liposomes overnight.

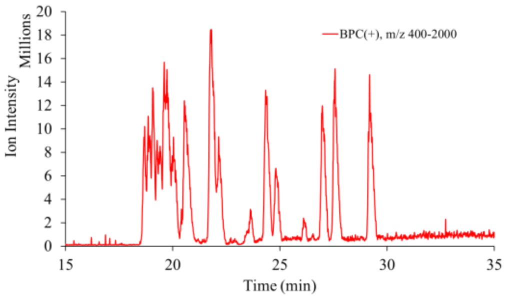

Figure S1. LC-MS of Env$_{37-44}$-Pos5 positional library. 4 mg mL^{-1} positional peptide libraries were injected to SunFire C8 50x2.1mm (Waters) column. Shimadzu Nexera XR UHPLC system and MS instrument Shimadzu LCMS-8045 triple quadrupole with ESI ionization and Q3 scan, monitoring at m/z 400-2000 were used.

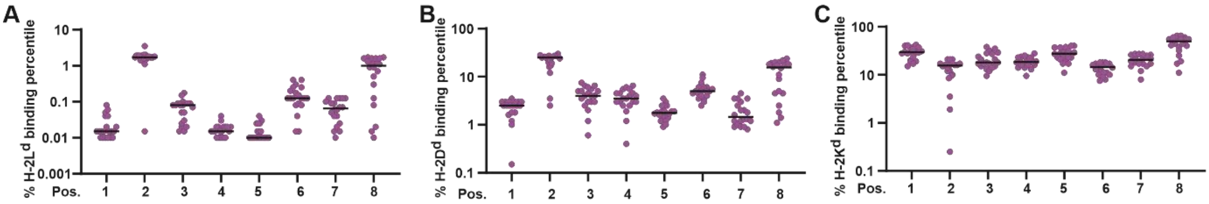

Figure S2. NetMHC binding percentile of Env$_{37\text{-}44}$ positional libraries. Peptide binding percentile to (**A**) H-2L^d, (**B**) H-2D^d and (**C**) H-2K^d.

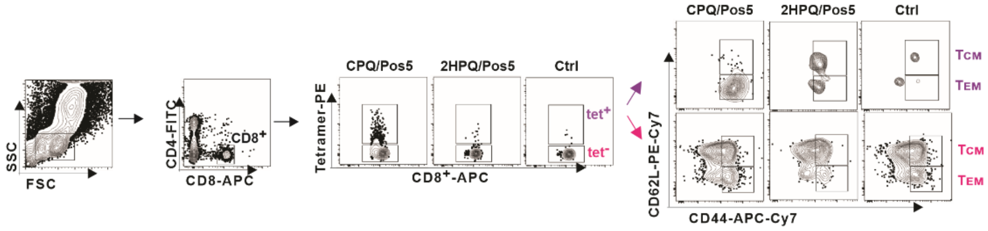

Figure S3. Flow cytometry gating of Env$_{37\text{-}44}$ tet$^+$ in CD8$^+$ T cells, T$_{EM}$ (CD62L$^-$ CD44$^+$) and T$_{CM}$ (CD62L$^+$ CD44$^+$) cells in the tet$^+$ or tet$^-$ T cells. Cells were first gated by CD8$^+$CD4$^-$. Env$_{37\text{-}44}$ tet$^+$ T cells and Env$_{37\text{-}44}$ tet$^-$ T cells were gated, then T$_{EM}$ and T$_{CM}$ in tet$^+$CD8$^+$ T cells and tet$^-$CD8$^+$ T cells were gated. Representative plots are shown from experiments with n=5 mice.

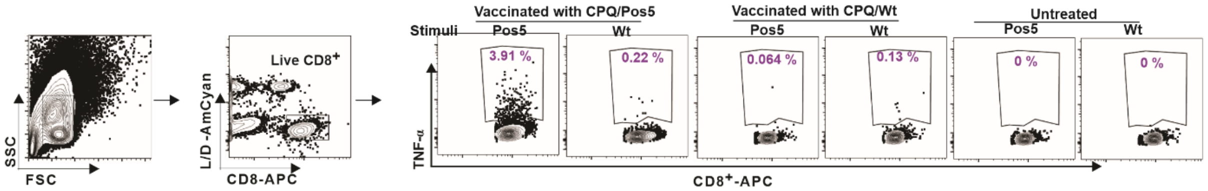

Figure S4. Flow cytometry gating of TNF-α producing CD8$^+$ T cells in spleen. Cells were first gated by CD8$^+$ L/D$^-$ then TNF-α producing CD8$^+$ T cells. Representative plots are shown from experiments with n=3 mice.

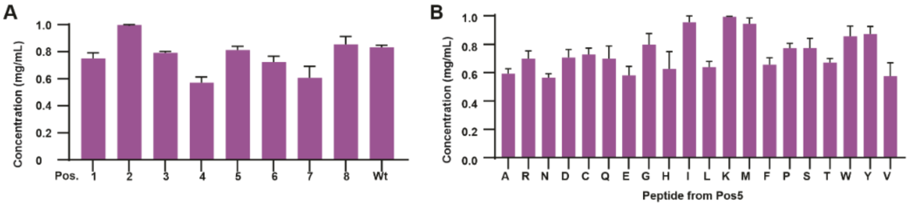

Figure S5. Concentration of stock peptide and peptide libraries measured by BCA assay. 1mg peptide powder was aliquoted to vials by Genscript, peptide libraries were directly dissolved in 1mL H_2O with concentration 1mg mL^{-1}. BCA assay was used to test the peptide concentration by comparing to a bovine serum albumin (BSA) standard curve. The concentration of peptide and peptide libraries varies from 0.6-1mg mL^{-1}. Since we assumed the concentration of peptides and peptide libraries were 1mg mL^{-1} when we prepared the vaccine, the actual vaccinated antigen dose might vary from 60-100% of the desired dose. Error bars show mean +/−std. dev. for n=3 triplicate experiments.

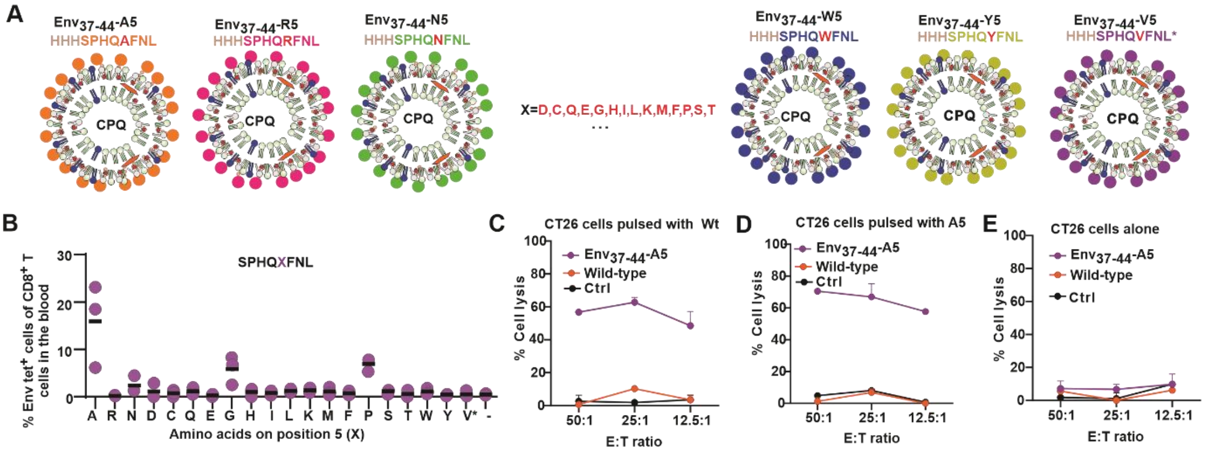

Figure S6. Interrogating the Env_{37-44}-Pos5 library identifies alanine substitution at position 5 of Env_{37-44} as an enhanced mimotope ("e-mimotope"). A) Scheme of using single peptide mimotope from Env_{37-44}-Pos5 as vaccine immunogen. BALB/c mice were vaccinated on day 0 & 7; blood was collected on day 14. Percentage of Env_{37-44} tet^{+} cells (**B**) in $CD8^{+}$ T cell population in blood. CT26 tumor cells with Env_{37-44} (**C**) or Env_{37-44}-A5 (**D**) pulse or without pulse (**E**) were served as target cells (T), splenocytes from untreated mice or mice vaccinated with CPQ/Env_{37-44}-A5 or CPQ/Env_{37-44} were stimulated with antigens and IL-2 then served as effector cells. Effector cells were incubated with target cells for 5 hours with an indicated E:T ratio. Error bars show mean +/−std. dev. for n=3 per group.

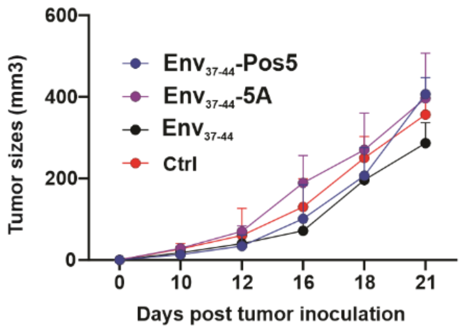

Figure S7. Either CPQ/Env_{37-44} -Pos5 or CPQ/ Env_{37-44} -5A delayed tumor growth in BALB/c mice. BALB/c mice were vaccinated on day 0 and day 7 and then inoculated with CT26 cells subcutaneously. Error bars show mean +/−std. dev. for n=5 mice per group.

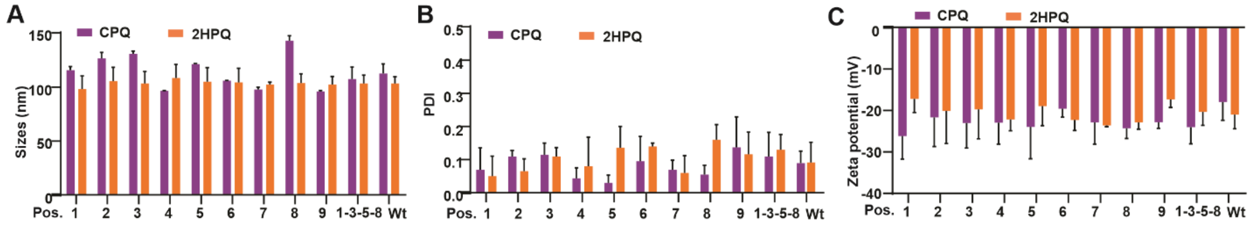

Figure S8. AH1 positional libraries formed nano particles with CPQ liposomes. Sizes (**A**), polydispersity (**B**) and zeta potential (**C**) of CPQ and 2HPQ liposomes after AH1 positional libraries and wild-type peptide binding. Error bars show mean +/−std. dev. for n=3 triplicate experiments.

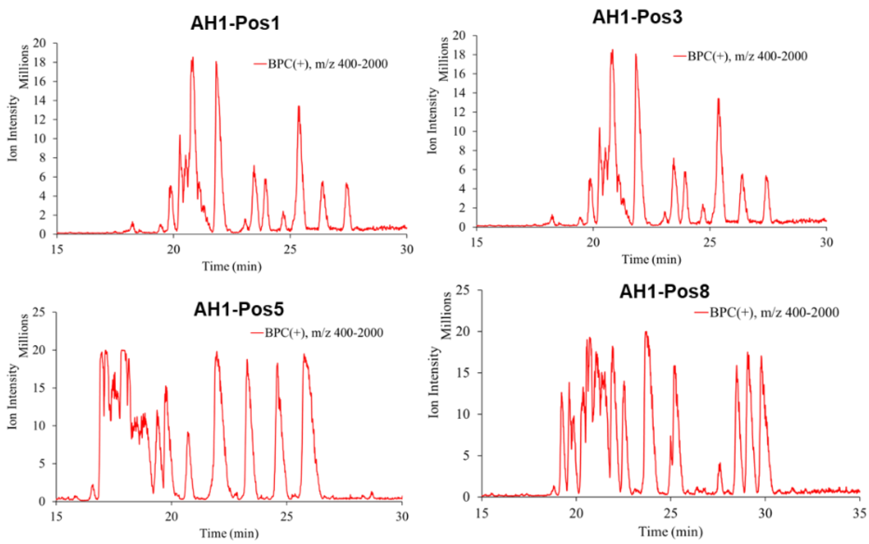

Figure S9. LC-MS of AH1 positional libraries. 4 mg mL^{-1} positional peptide libraries were injected to SunFire C8 50x2.1mm (Waters) column. Shimadzu Nexera XR UHPLC system and MS instrument Shimadzu LCMS-8045 triple quadrupole with ESI ionization and Q3 scan, monitoring at m/z 400-2000 were used.

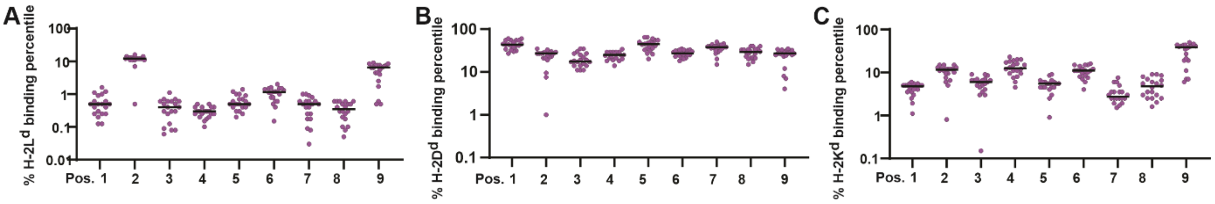

Figure S10. NetMHC binding percentile of AH1 positional libraries. Peptide binding percentile to (**A**) H-2L^d, (**B**) H-2D^d and (**C**) H-2K^d.

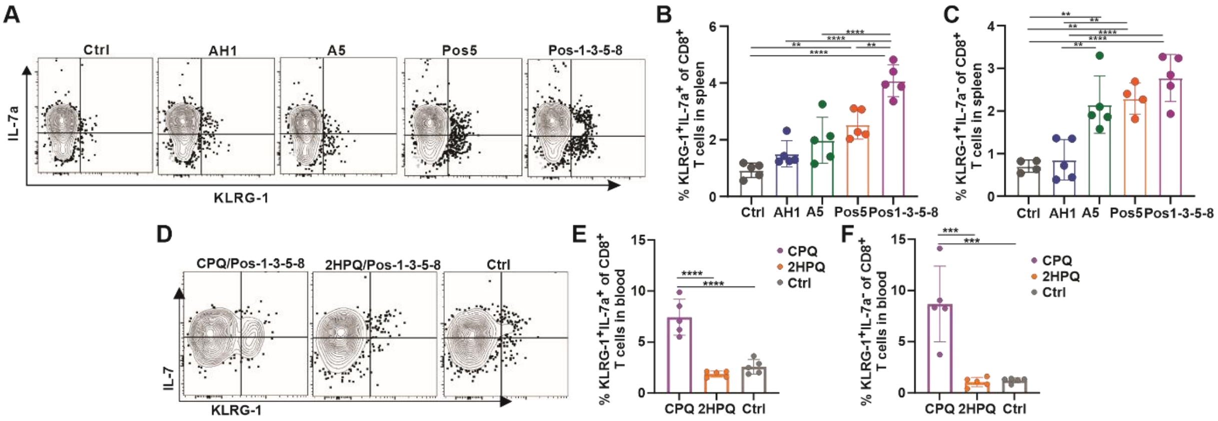

Figure S11. BALB/c mice were inoculated with CT26 cells subcutaneously on day 0 and then untreated or injected with vaccine on days 8 &15. Splenocytes were collected on day 23. Flow cytometry gating (**A**) and percentage of KLRG-1$^+$IL7-a$^+$ (**B**) and KLRG-1$^+$IL7-a$^-$ cells (**C**) in CD8$^+$ T cell population. BALB/c mice were inoculated with CT26 cells subcutaneously on day 0; then the vaccine was given 12&19 days post-tumor inoculation. Blood was collected on day 26. Flow cytometry gating **(D)** and percentage of KLRG-1$^+$IL7-a$^+$ (**E**) and KLRG-1$^+$IL7-a$^-$ cells (**F**) in CD8$^+$ T cell population. Error bars show mean +/−std. dev. for n=5 mice. ** $p < 0.01$, *** $p < 0.001$, and **** $p < 0.0001$, analyzed one-way ANOVA with Bonferroni multiple comparisons post-test.

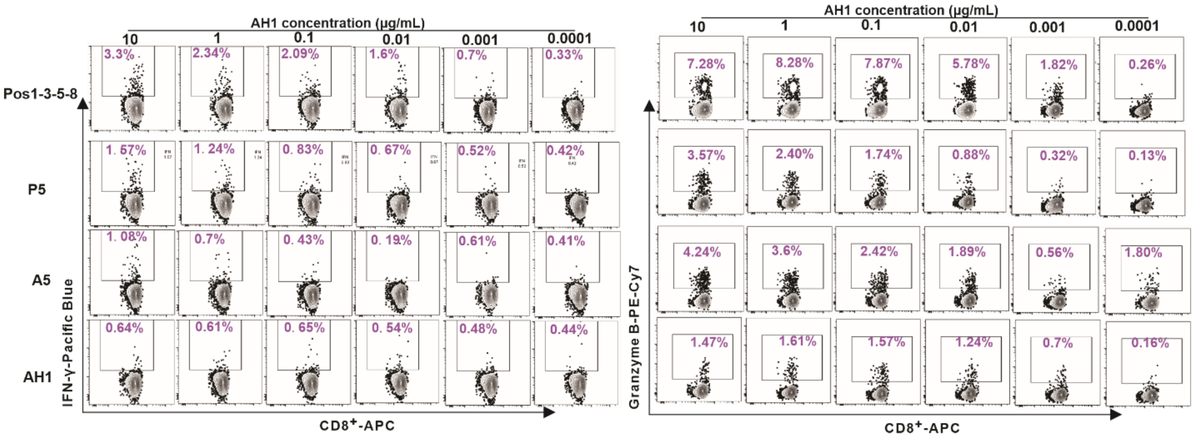

Figure S12. Flow cytometry gating of IFN-γ-producing cells and granzyme B-producing cells in the CD8+ T cell population. BALB/c mice were inoculated with CT26 cells subcutaneously on day 0 and then untreated or injected with vaccine on days 8 &15. Splenocytes were collected on day 23 and stimulated with different concentrations of the wild-type AH1 peptide,followed by analysis of IFN-γ and granzyme B expression by intracellular staining. Representative plots are shown from experiments with n=5 mice.

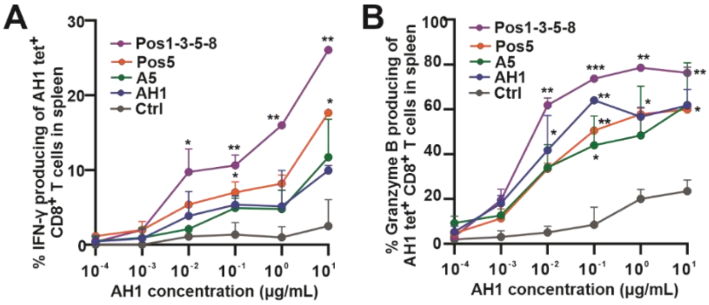

Figure S13. A greater frequency of AH1-specific CD8+ T cells generated by the positional library vaccine produced cytokines compared to single e-mimotope vaccine. BALB/c mice were inoculated with CT26 cells subcutaneously on day 0 and then remained untreated or injected with vaccine on days 8 & 15. Splenocytes were collected on day 23 and stimulated with different concentrations of the wild-type AH1 peptide, followed by analysis of IFN-γ and granzyme B expression by intracellular staining. Percentage of IFN-γ-producing cells (**A**) and granzyme B-producing cells (**B**) in the AH1-tet+CD8+ T cell population. * $p < 0.05$, ** $p < 0.01$, and *** $p < 0.001$ analyzed by one-way ANOVA with Dunnett comparisons post-test. Error bars show mean +/−std. dev. for n=5 mice. Asterisks indicate statistically significant differences between indicated group and control group with indicated AH1 concentration.

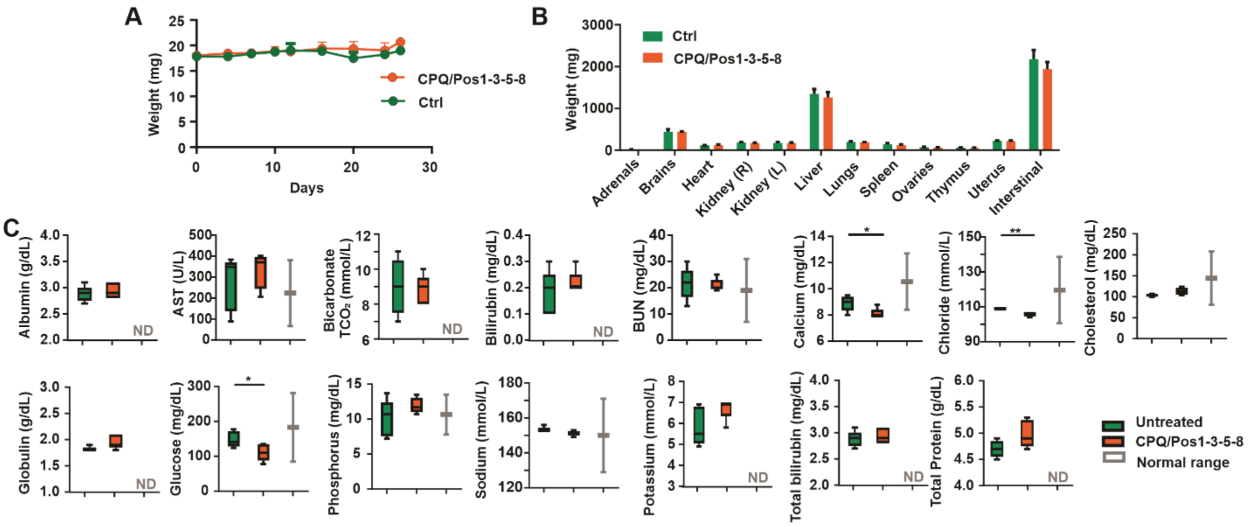

Figure S14. Safety of CPQ/Pos1-3-5-8 vaccine. BALB/c mice were inoculated with CT26 cells subcutaneously on day 0; then vaccine was given 12&19 days post-tumor inoculation, blood and organs were collected 26 days post- tumor inoculation. **A**) Weight of mice. **B**) Weight of organs from mice. **C**) Serum markers with their general description as follows: albumin, AST (aspartate aminotransferase), bicarbonate, bilirubin, BUN (blood urea nitrogen), calcium, chloride, cholesterol, globulin, glucose, phosphorus, sodium, potassium, total bilirubin, total protein. Values show mean +/- std. dev for n=5 mice per group. "ND"; no data provided for normal range. Data show box-and-whiskers plots, the line represents the median, the whiskers show the data range and the box shows the interquartile range. * $p < 0.05$, ** $p < 0.01$, analyzed by two-tailed unpaired Student's *t*-test.

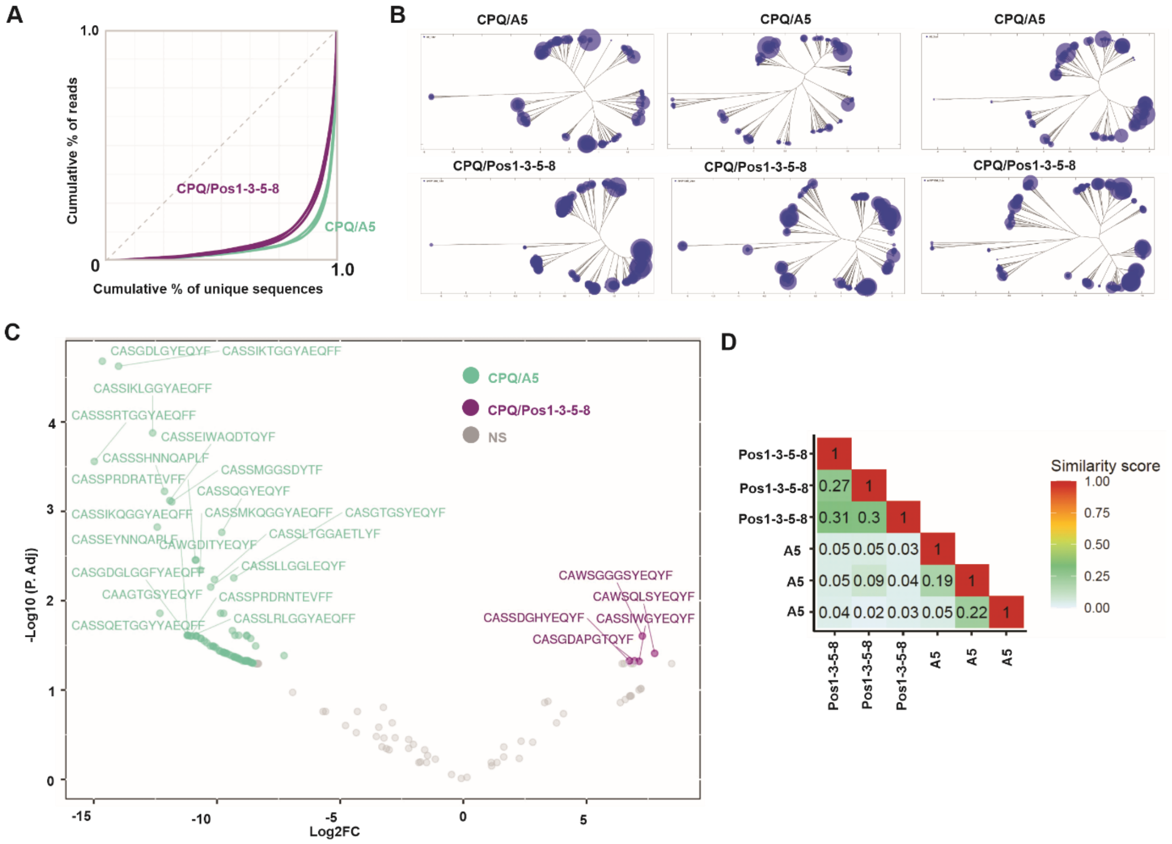

Figure S15. Effective vaccine therapy induces expansion of highly diverse tumor-specific CD8$^+$ T cell repertoires. Mice were vaccinated with CPQ/A5 or CPQ/Pos1-3-5-8 on day 0 &7, tet$^+$CD8$^+$ T cells were sorted from splenocytes on day 14, DNA were extracted for ImmunoSEQ immune profiling system at the survey level. **A**) Lorenz curve of CPQ/A5 and

CPQ/Pos1-3-5-8. **B**) ImmunoMAP analyses of tet$^+$CD8$^+$ T cells from CPQ/A5 and CPQ/Pos1-3-5-8 vaccinated mice. **C**) Volcano plot depicting differentially abundant clonotypes observed from CPQ/A5 and CPQ/Pos1-3-5-8 vaccinated mice. (**D**) Similarity matrix of TCR repertoires from tet$^+$CD8$^+$ T cells from CPQ/A5 and CPQ/Pos1-3-5-8 vaccinated mice.

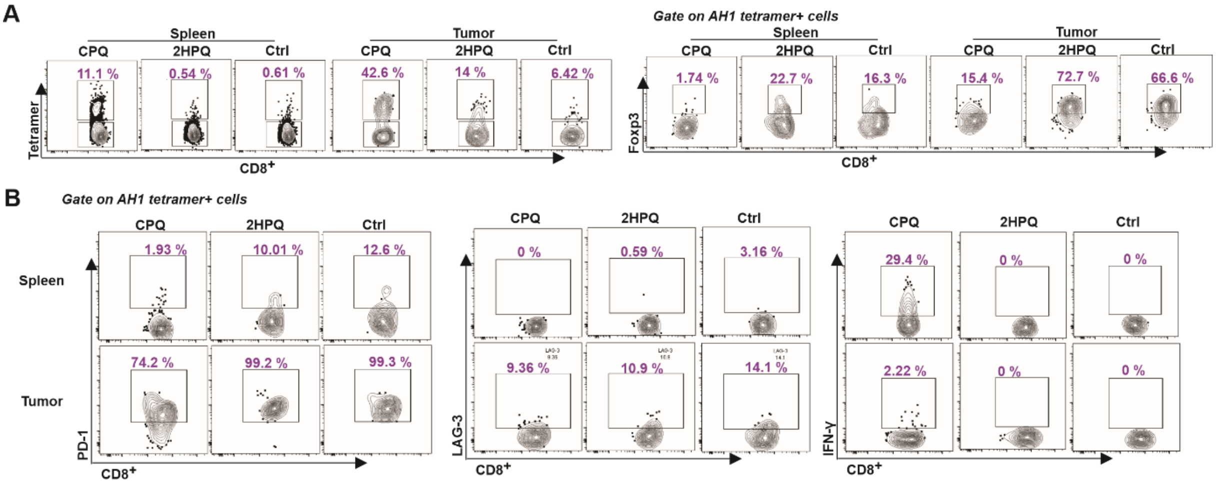

Figure S16. Flow cytometry gating of Treg (Foxp3$^+$) and PD-1$^+$, LAG3$^+$, IFN-γ$^+$ T cells in the AH1 tet$^+$ T cell population. BALB/c mice were inoculated with CT26 cells subcutaneously on day 0; then vaccine was given 12&19 days post-tumor inoculation, spleen and tumor tissue were collected 26 days post- tumor inoculation for CD8$^+$ T cell analysis. **A**) Flow cytometry gating of AH1 tet$^+$ cells in the CD8$^+$ T cell population (left), foxp3$^+$ cells in the tet$^+$CD8$^+$ T cell population. **B**) flow cytometry gating of PD-1$^+$, LAG-3$^+$ or IFN-γ$^+$ cells within the tet$^+$CD8$^+$ T cells. Representative plots are shown from experiments with n=5 mice.

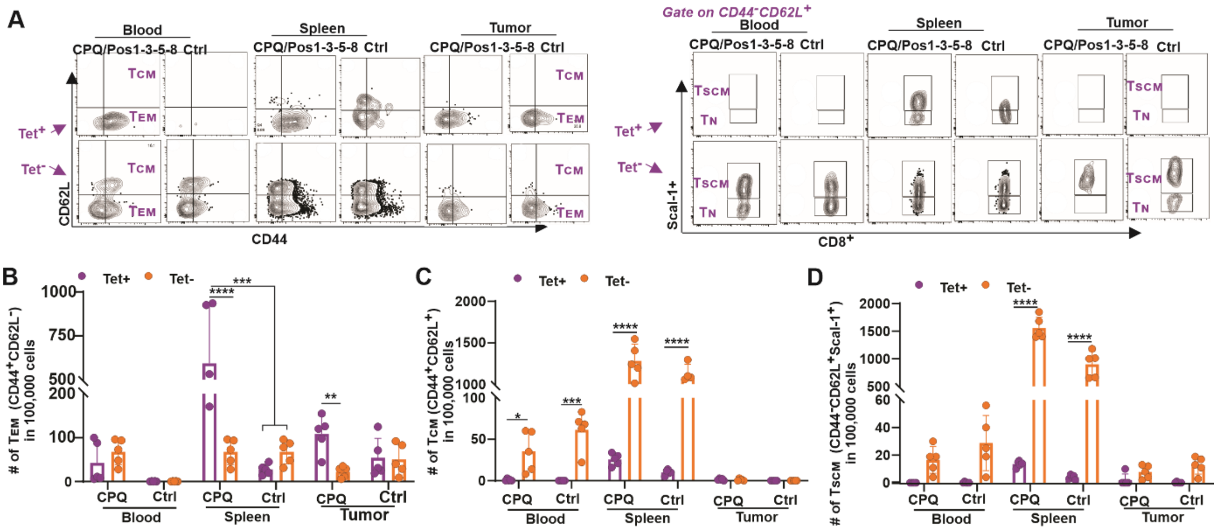

Figure S17. Memory T cell phenotyping. BALB/c mice were inoculated with CT26 cells subcutaneously on day 0; then vaccine was given 12&19 days post-tumor inoculation, blood, spleen and tumor were collected 26 days post- tumor inoculation for CD8$^+$ T cell analysis. **A**) Flow cytometry gating of T_{EM} (CD44$^+$CD62L$^-$), T_{CM}(CD44$^+$CD62L$^+$) cells and T_{SCM} (CD44$^-$CD62L$^+$Scal-1$^+$) in the tet$^+$ CD8$^+$ or tet$^-$ CD8$^+$ T cell population. Number of T_{EM} (**B**), T_{CM} (**C**) and T_{SCM} (D) in 100,000 cells in blood, spleen and tumor. ** $p < 0.01$, and *** $p < 0.001$ and **** $p < 0.0001$, analyzed by one-way ANOVA with Tukey multiple comparisons post-test. Representative plots are shown from experiments with n=5 mice.

Table S1. LC-MS of positional libraries

Positional library	Peak Time Start (min)	m/z (charge)	X=	Theoretical mass	Peak areas
$En_{37\text{-}44}$-Pos5	18.562	1382 (1+), 692 (2+), 461 (3+)	K	1381	63908023
$En_{37\text{-}44}$-Pos5	18.782	1368 (1+), 684 (2+), 457 (3+)	N	1367	91034033
$En_{37\text{-}44}$-Pos5	18.97	1391 (1+), 696 (2+), 464 (3+)	H	1390	127962133
$En_{37\text{-}44}$-Pos5	19.211	1341 (1+), 671 (2+), 448 (3+)	S	1340	107267716
$En_{37\text{-}44}$-Pos5	19.211	1382 (1+), 691 (2+), 461 (3+)	Q	1381	47631223
$En_{37\text{-}44}$-Pos5	19.211	1410 (1+), 706 (2+), 471 (3+)	R	1409	67355896
$En_{37\text{-}44}$-Pos5	19.538	1311 (1+), 656 (2+), 438 (3+)	G	1310	163288336
$En_{37\text{-}44}$-Pos5	19.538	1369 (1+), 685 (2+), 457 (3+)	D	1368	263630747
$En_{37\text{-}44}$-Pos5	19.95	1401 (1+), 701 (2+), 468 (3+)	F	1400	146132070
$En_{37\text{-}44}$-Pos5	20.428	1325 (1+), 663 (2+), 442 (3+)	A	1324	155,755,912
$En_{37\text{-}44}$-Pos5	20.428	1383 (1+), 692 (2+), 462 (3+)	E	1382	182549603
$En_{37\text{-}44}$-Pos5	21.645	1357 (1+), 679 (2+), 453 (3+)	C	1356	290,882,708
$En_{37\text{-}44}$-Pos5	22.053	1351 (1+), 676 (2+), 451 (3+)	P	1350	127889034
$En_{37\text{-}44}$-Pos5	23.372	1353 (1+), 677 (2+), 452 (3+)	V	1,352	31907686
$En_{37\text{-}44}$-Pos5	23.372	678 (2+)	T	1354	9682905
$En_{37\text{-}44}$-Pos5	24.144	1417 (1+), 709 (2+), 473 (3+)	Y	1416	184219831
$En_{37\text{-}44}$-Pos5	24.658	1385 (1+), 693 (2+), 462 (3+)	M	1384	76445569
$En_{37\text{-}44}$-Pos5	25.993	1367 (1+), 684 (2+), 456 (3+)	I, L	1366	19483118
$En_{37\text{-}44}$-Pos5	26.851	1367 (1+), 684 (2+), 456 (3+)	I, L	1366	144202744
$En_{37\text{-}44}$-Pos5	27.382	1401 (1+), 701 (2+), 468 (3+)	F	1400	189682935
$En_{37\text{-}44}$-Pos5	29.04	1440 (1+), 721 (2+), 481 (3+)	W	1439	153300993
AH1-Pos1	19.766	791(2+)	K	1580	35547329
AH1-Pos1	19.766	795(2+)	H	1588	92051659
AH1-Pos1	19.766	1581 (1+), 791(2+), 527 (3+)	E	1580	44592330
AH1-Pos1	20.15	763(2+)	A	1523	25150497
AH1-Pos1	20.15	1510 (1+), 755 (2+), 504 (3+)	G	1509	112660467
AH1-Pos1	20.15	791(2+)	Q	1579	53824617
AH1-Pos1	20.15	805(2+)	R	1608	14497409
AH1-Pos1	20.15	771(2+)	S	1539	34471761
AH1-Pos1	20.15	777(2+)	V	1551	891411
AH1-Pos1	20.15	1555 (1+), 778 (2+), 519 (3+)	C	1555	119284960
AH1-Pos1	22.947	1552 (1+), 776 (2+), 518 (3+)	T	1553	9366602
AH1-Pos1	23.331	1615 (1+), 808 (2+), 539 (3+)	Y	1615	40892550
AH1-Pos1	23.715	1583 (1+), 792 (2+), 528 (3+)	M	1583	27104761
AH1-Pos1	24.487	1565 (1+), 783 (2+), 522 (3+)	N	1565	135462
AH1-Pos1	25.144	1565 (1+), 783 (2+), 523 (3+)	L, I	1565, 1564	182607891
AH1-Pos1	25.144	784(2+)	D	1566	71234179
AH1-Pos1	26.132	1599 (1+), 800 (2+), 534 (3+)	F	1599	32280677
AH1-Pos1	27.174	1639 (1+), 820 (2+), 547 (3+)	W	1638	58459667
AH1-Pos3	19.489	1581 (1+), 791 (2+), 572 (3+)	K	1580	36246274
AH1-Pos3	19.489	1590 (1+), 795 (2+), 530 (3+)	H	1588	52976797

AH1-Pos3	19.983	1509 (1+), 755 (2+), 504 (3+)	G	1509	38221048
AH1-Pos3	19.983	1566 (1+), 784 (2+), 523 (3+)	D	1,566	49355197
AH1-Pos3	19.983	1608 (1+), 805 (2+), 537 (3+)	R	1608	37579640
AH1-Pos3	20.477	1539 (1+), 770 (2+), 514 (3+)	S	1539	23905901
AH1-Pos3	20.477	1580 (1+), 791 (2+), 527 (3+)	Q	1579	28975203
AH1-Pos3	20.477	1599 (1+), 800 (2+), 534 (3+)	F	1599	8152448
AH1-Pos3	20.918	1523 (1+), 762 (2+), 508 (3+)	A	1523	51684365
AH1-Pos3	20.918	1549 (1+), 775 (2+), 517 (3+)	P	1549	12776902
AH1-Pos3	20.918	1567 (1+), 784 (2+), 523 (3+)	D	1566	157540361
AH1-Pos3	20.918	1581 (1+), 791 (2+), 528 (3+)	E	1580	77398174
AH1-Pos3	22.016	779(2+)	C	1555,	105995568
AH1-Pos3	22.016	1555 (1+), 778 (2+), 519 (3+)	T	1553	221084116
AH1-Pos3	22.837	777(2+)	T	1553	4300892
AH1-Pos3	22.837	1551 (1+), 776 (2+), 518 (3+)	V	1,551	19200301
AH1-Pos3	23.388	1615 (1+), 808 (2+), 539 (3+)	Y	1615	49876713
AH1-Pos3	23.388	1584 (1+), 792 (2+), 529 (3+)	M	1583	14222197
AH1-Pos3	24.54	1566 (1+), 783 (2+), 523 (3+)	N	1,565	6122778
AH1-Pos3	24.54	1565 (1+), 783 (2+), 523 (3+)	I	1564	86043933
AH1-Pos3	24.54	784 (2+)	L	1564	71234179
AH1-Pos3	27.725	1638 (1+), 820 (2+), 547 (3+)	W	1638	118413841
AH1-Pos5	16.855	1497 (1+), 749 (2+), 500 (3+)	G	1497	123300827
AH1-Pos5	16.855	1527 (1+), 764 (2+), 510 (3+)	S	1528	
AH1-Pos5	16.855	785(2+)	K	1569	192556994
AH1-Pos5	16.855	785(2+)	Q	1569	196250189
AH1-Pos5	16.855	1577 (1+), 789 (2+), 526 (3+)	H	1577	128358085
AH1-Pos5	16.855	1587 (1+), 794 (2+), 530 (3+)	F	1588	221666366
AH1-Pos5	16.855	1596 (1+), 799 (2+), 533 (3+)	R	1597	157489852
AH1-Pos5	18.337	1511 (1+), 756 (2+), 504 (3+)	A	1511	87040717
AH1-Pos5	18.337	1527 (1+), 764 (2+), 510 (3+)	S	1528	78168203
AH1-Pos5	18.337	1541 (1+), 771 (2+), 514 (3+)	W	1,540	5208793
AH1-Pos5	18.337	779(2+)	D	1,555	85580867
AH1-Pos5	18.337	1555 (1+), 778 (2+), 519 (3+)	N	1,554	147850158
AH1-Pos5	18.337	1569 (1+), 785 (2+), 524 (3+)	E	1,568	29158895
AH1-Pos5	19.215	1537 (1+), 769 (2+), 513 (3+)	P	1537	66235240
AH1-Pos5	19.709	773	T	1542	89665213
AH1-Pos5	19.709	1543 (1+), 772 (2+), 515 (3+)	C	1543	205657263
AH1-Pos5	20.534	1539 (1+), 770 (2+), 514 (3+)	V	1539	103906082
AH1-Pos5	20.534	787 (2+)	M	1571	3114580
AH1-Pos5	21.743	1603 (1+), 802 (2+), 535 (3+)	Y	1604	191721914
AH1-Pos5	23.058	1553 (1+), 777 (2+), 518 (3+)	I, L	1,553	41309976
AH1-Pos5	24.43	1587 (1+), 794 (2+), 530 (3+)	F	1588	138967104
AH1-Pos5	25.581	1626 (1+), 814 (2+), 543 (3+)	W	1627	477275715
AH1-Pos8	18.995	1539 (1+), 770 (2+), 514 (3+)	K	1,539	45312581
AH1-Pos8	19.599	1548 (1+), 775 (2+), 517 (3+)	H	1548	119661122
AH1-Pos8	19.599	1567 (1+), 784 (2+), 523 (3+)	R	1567	57101274
AH1-Pos8	20.203	1468 (1+), 735 (2+), 490 (3+)	G	1468	210562395
AH1-Pos8	20.203	1498 (1+), 750 (2+), 500 (3+)	S	1498	148067561
AH1-Pos8	20.203	1512 (1+), 757 (2+), 505 (3+)	T	1511	85624310

AH1-Pos8	20.203	1526 (1+), 763 (2+), 509 (3+)	N	1,525	312239940
AH1-Pos8	20.203	772(2+)	M	1542	35862517
AH1-Pos8	20.203	1539 (1+), 770 (2+), 514 (3+)	E	1,540	180611625
AH1-Pos8	20.203	1558 (1+), 780 (2+), 520 (3+)	F	1558	263732949
AH1-Pos8	21.849	1482 (1+), 742 (2+), 495 (3+)	A	1482	274748057
AH1-Pos8	21.849	1508 (1+), 755 (2+), 503 (3+)	P	1508	180208011
AH1-Pos8	21.849	1514 (1+), 758 (2+), 505 (3+)	C	1514	433965311
AH1-Pos8	24.871	1574 (1+), 488 (2+), 525 (3+)	Y	1574	256520687
AH1-Pos8	24.871	1510 (1+), 756 (2+), 504 (3+)	V	1510	43455764
AH1-Pos8	27.394	1525 (1+), 763 (2+), 509 (3+)	I, L	1,524	17379004
AH1-Pos8	28.435	764(2+)	D	1526	26979268
AH1-Pos8	28.435	1524 (1+), 763 (2+), 509 (3+)	I, L	1,524	185028475
AH1-Pos8	29.04	1558 (1+), 780 (2+), 520 (3+)	F	1558	278190170
AH1-Pos8	29.864	1597 (1+), 799 (2+), 533 (3+)	W	1597	152600419

Table S2. Env$_{37-44}$-Pos5 peptides

Name	Sequence	MHC allele	NetMHC percentile	HPLC purity	Observed MW	Theoretical MW
Env$_{37-44}$-A5	HHHSPHQAFNL	H2-L^d	0.2	81.8	1324.2	1324.41
Env$_{37-44}$-R5	HHHSPHQRFNL	H2-L^d	1.4	98.2	1408.8	1409.52
Env$_{37-44}$-N5	HHHSPHQNFNL	H2-L^d	0.4	91	1367.1	1367.44
Env$_{37-44}$-D5	HHHSPHQDFNL	H2-L^d	1.2	91.6	1368.3	1368.42
Env$_{37-44}$-C5	HHHSPHQCFNL	H2-L^d	0.25	82.4	1356.3	1356.48
Env$_{37-44}$-Q5	HHHSPHQQFNL	H2-L^d	0.5	76.2	1381.2	1381.46
Env$_{37-44}$-E5	HHHSPHQEFNL	H2-L^d	1	84.9	1382.1	1382.45
Env$_{37-44}$-G5	HHHSPHQGFNL	H2-L^d	0.6	89.6	1310.1	1310.39
Env$_{37-44}$-H5	HHHSPHQHFNL	H2-L^d	0.5	81.9	1390.2	1390.47
Env$_{37-44}$-I5	HHHSPHQIFNL	H2-L^d	0.4	85.6	1366.2	1366.49
Env$_{37-44}$-L5	HHHSPHQLFNL	H2-L^d	0.7	86.1	1366.2	1366.49
Env$_{37-44}$-K5	HHHSPHQKFNL	H2-L^d	0.9	98.7	1381.2	1381.51
Env$_{37-44}$-M5	HHHSPHQMFNL	H2-L^d	0.3	82.1	1384.2	1384.53
Env$_{37-44}$-F5	HHHSPHQFFNL	H2-L^d	0.6	89.3	1400.1	1400.51
Env$_{37-44}$-P5	HHHSPHQPFNL	H2-L^d	1	81.8	1350.3	1350.45
Env$_{37-44}$-S5	HHHSPHQSFNL	H2-L^d	0.3	88.1	1340.1	1340.41
Env$_{37-44}$-T5	HHHSPHQTFNL	H2-L^d	0.7	72.1	1354.2	1354.44
Env$_{37-44}$-W5	HHHSPHQWFNL	H2-L^d	0.5	88.7	1439.1	1439.55
Env$_{37-44}$-Y5	HHHSPHQYFNL	H2-L^d	0.4	77.7	1416	1416.51
Env$_{37-44}$-V5	HHHSPHQVFNL	H2-L^d	0.5	95.2	1351.8	1352.47
